# Bioethics of somatic gene therapy: what do we know so far?

**DOI:** 10.1080/03007995.2023.2257600

**Published:** 2023-10-10

**Authors:** Paola Buedo, Alahi Bianchini, Katarzyna Klas, Marcin Waligora

**Affiliations:** a Research Ethics in Medicine Study Group (REMEDY), Jagiellonian University Medical College, Krakow, Poland; b Instituto de Investigaciones Jurídicas y Sociales Ambrosio Lucas Gioja, Universidad de Buenos Aires, Buenos Aires, Argentina - Programa de Bioética, FLACSO Argentina, Buenos Aires, Argentina

**Keywords:** Gene therapy, somatic cells, bioethics, risk assessment, ethics, social impact

## Abstract

**Objective:**

To provide a systematic overview of bioethical debate on somatic gene therapy as documented in the scientific literature.

**Methods:**

We performed a systematic review of reasons, following Strech and Sofaer approach, which is a method to systematically identify and classify arguments (reasons) used in the scientific literature. We identified 217 eligible publications retrieved from PubMed, Lilacs, PhilPapers, and Google Scholar. A meta-synthesis was performed to analyze the data.

**Results:**

We extracted 189 arguments. Arguments were grouped into 23 categories. Twelve categories were classified as research-related, including the risk/benefit ratio, priorities and limits, informed consent, review, and monitoring. Eleven were classified as society-related, including population impact, human identity, public perception, human health.

**Conclusion:**

Our study provides a database of existing challenges and arguments of somatic gene therapy and may serve as the basis of normative analysis. By presenting collected arguments, we contribute to the discussion about the ethics and social dimensions of somatic gene therapy.

## Introduction

Gene therapy is defined as a technique that modifies a person’s genes for therapeutic purposes^1,2^. Introducing a new copy of an exogenous gene^3^, replacing or inactivating a gene^1,2^ or editing genes^4^ are some techniques used in the gene therapy field. According to the cellular target, gene therapy can also be classified in somatic and germline gene therapy. Somatic gene therapy is oriented to treat only the person receiving the therapy, whereas germline gene therapy treats the person, and the results of this procedure can be inherited by his/her descendants^5,6^. Most current research focuses mainly on somatic gene therapy^7^.

Somatic gene therapy is a promising approach that could provide treatment options for many diseases^8^. There are many preclinical and clinical studies that evaluate the therapeutic potential of interventions in human genes^5,7^. For example, in relation to curing various types of cancer (meningiomas and spinal cord, gastrointestinal, breast, etc.), genetic disorders (such as thalassemia or severe combined immunodeficiency), infectious diseases (such as HIV or hepatitis), cardiovascular diseases (such as coronary artery disease or ischemia), among others^7^.

The latest and more innovative techniques used for gene therapy are cutting-edge molecular tools that correct errors within genes, like CRISPR-CAS9, which is a simple, precise and rapid genome editing technology. Replacing, silencing or inserting an entire gene is now a kind of conventional somatic gene therapy after the emergence of CRISPR^4^.

Conventional somatic gene therapy (i.e. non-editing somatic gene therapy) currently gets less attention in the discussion about ethics implications since the debate of CRISPR technologies^9,10^. However, complex techniques or more invasive ones – such as CRISPR – should not distract us from the important ethical debate and unresolved questions. Somatic gene therapy will soon transform into a massive scale medical procedure: thus the unresolved ethical challenges need to be re-examined^5,6,11–13^.

In the following, we identify, categorize and analyze arguments on bioethical challenges of conventional somatic gene therapy. The aim of our study is to provide a systematic overview of the arguments used in the discussion about human gene therapy in somatic cells using conventional techniques that are documented in scientific literature.

## Methods

We performed a systematic review of reasons^14^, following Strech and Sofaer approach, which is a method to systematically identify and classify arguments (reasons) used in the scientific literature. We described the methods in detail below. A meta-synthesis^15,16^ was performed to analyze all data. The study protocol was prospectively registered on Open Science Framework (see https://osf.io/fxuwj) (S1 Appendix).

### Eligibility criteria

Publications were eligible if they had focused on somatic gene therapy with clear therapeutic goals and discussed reasons or premises about acceptability, importance, value, morality, ethics, or bioethics. We included articles in English or Spanish and the following types: (i) normative articles focusing on somatic gene transfer/therapy and its ethical/bioethical aspects; (ii) articles focusing on public perception or use of somatic gene transfer/therapy; (iii) articles focusing on professionals and researchers about ethical aspects of somatic gene transfer/therapy; (iv) narrative reviews, editorials, commentaries, opinions, letters, guidelines and policy recommendations. To make the search feasible, we excluded articles focused entirely on the ethics/bioethics of germline gene transfer or genome editing because it was out of the scope of our study; articles that focused on intrauterine, fetal, or prenatal gene transfer because we understand that this particular context could raise other ethical issues apart from those of the gene therapy itself; reports of interventional studies of gene transfer/therapy, as our objective is the ethical approach of the technique; articles from press and books, book chapters, comments on books, and congress abstracts/posters.

### Search strategy

We performed the search in PubMed, Lilacs, PhilPapers, and Google Scholar on 26 July 2021. We chose these databases because they cover a wide range of biomedical and philosophical publications from all over the world. Choosing Lilacs, which is the most important Latin American database, allowed us to be sensitive to cultural or otherwise region-dependent differences. We performed the search without time restrictions. The only restriction that we used was in Google Scholar database because of the large number of articles that the search retrieved. We decided to use the first 100 hits^17^. The search strategy for each database is presented in the Supplementary Material section (S2 Appendix).

### Selection process

Based on the pre-specified eligibility criteria, PB, AB, and KK independently screened the search results in two stages: first, titles and abstracts and second, the full texts. At each stage, we independently double screened all references. In case of any disagreements, a discursive consensus was reached.

### Data extraction

The selected articles were analyzed using three prospectively designed data extraction documents (S3 Appendix). Contextual data from the included articles i.e. year, journal and language of publication, article type, field (according to Journal Citation Reports (JCR); if the journal were not indexed, we classified the journal field according to the journal scope based on its website), number, affiliation, and country of authors, were obtained using the first data extraction document. Subsequently, all arguments related to the bioethics of human gene therapy were extracted and organized in another data extraction document, including the argument extracted and the number of references. At this stage, we used the constant comparative method (CCM)^15^. Before starting the extraction, researchers were trained in CCM.

### Identification of codes and themes

We grouped the extracted arguments into categories related to a certain topic^18^. The formulation of the categories was an iterative process. Categories are not supposed to be exhaustive or exclusive. There may be some arguments that correspond to two or more categories. However, we decided to include each argument only in one category to make our results more comprehensive. We discussed the categories several times among all researchers to find the best match for each argument. The categories were also grouped into two broad themes.

### Quality appraisal

As described in the “Selection process” section, the article screening and extraction process was carried out independently by three researchers, who have different professional backgrounds (pharmacist, medical doctor and philosopher, with post-graduate studies in bioethics). The multidisciplinary approach was important to consider different points of view and ways of thinking. Screening, extraction, and category formulation were supervised by a bioethics expert (MW).

### Data reporting

The data report follows the PRISMA Ethics – Reporting guideline for systematic reviews on ethics literature: development, explanations and examples^19^. The PRISMA-Ethics Reporting Guideline of this review can be found in the Supplementary Material section (S4 Appendix).

## Results

### Publication selection process

**Figure 1.**
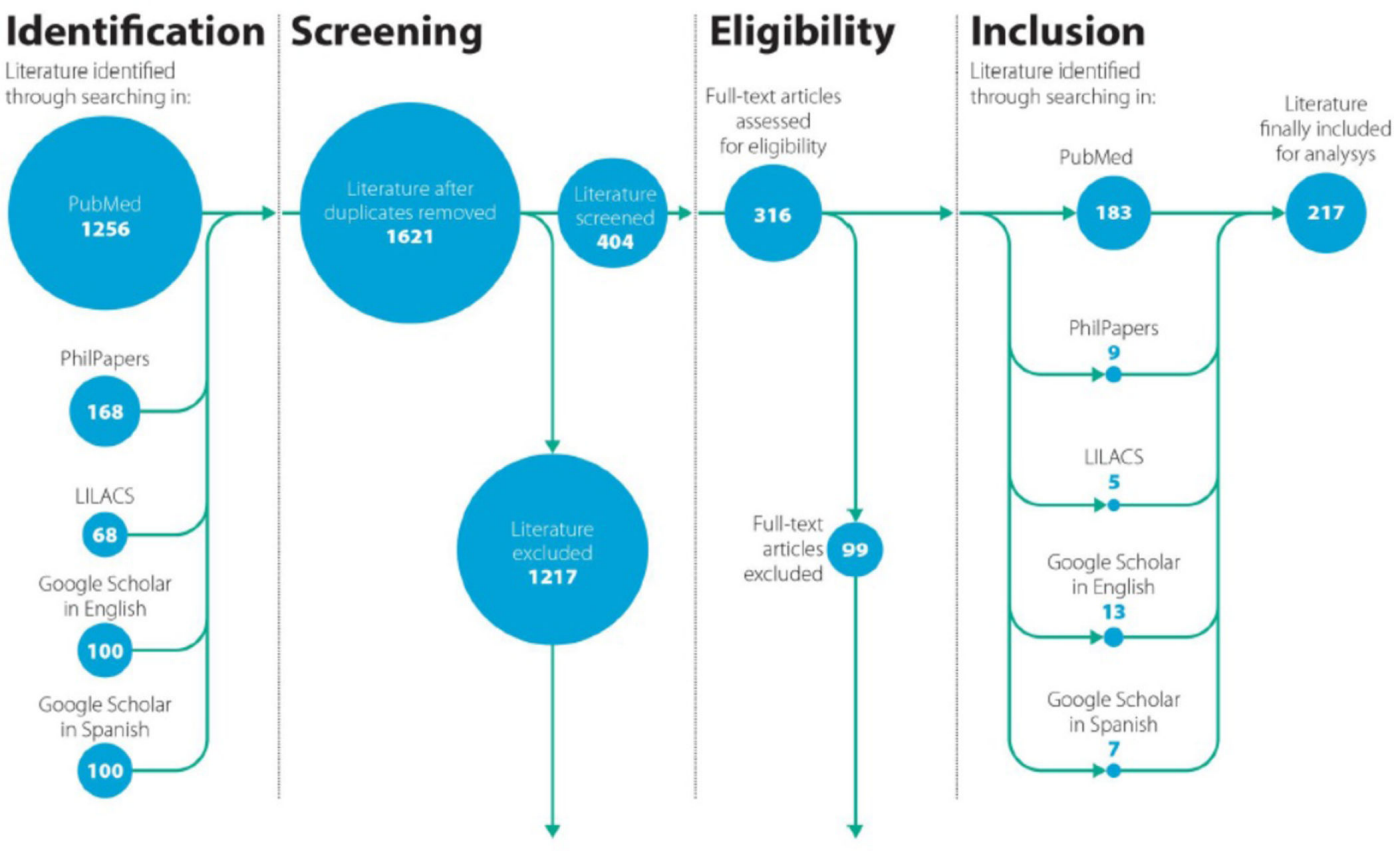
The PRISMA flow diagram of the study selection process.

The systematic search yielded 1701 results. Removal of duplications left 1621 references. Title/Abstract screening resulted in 404 potentially eligible documents. After full text screening, we included 217 articles that met the eligibility criteria (Figure 1). The cohort of included articles is listed in Table 1, and with full details in the Supplementary Material section (S5 Appendix).

### Characteristics of publications

Of the 217 articles included, 206 (94,9%) were published in English and 11 (5,1%) in Spanish. The earliest came from 1972, the last from 2020. The most prominent types of publications were reviews and theoretical/conceptual papers. Almost half of the authors (46,7%) of all selected publications were from the US, followed by Canada (12,4%) and the UK (9,8%). The journal that published the highest number of articles was Human Gene Therapy (*n*= 36; 16,6%). The largest number of articles were from the academic field of bioethics and genetics. More details can be found in the Supplementary Material section (S6 Appendix).

### Results of syntheses

**Figure 2.**
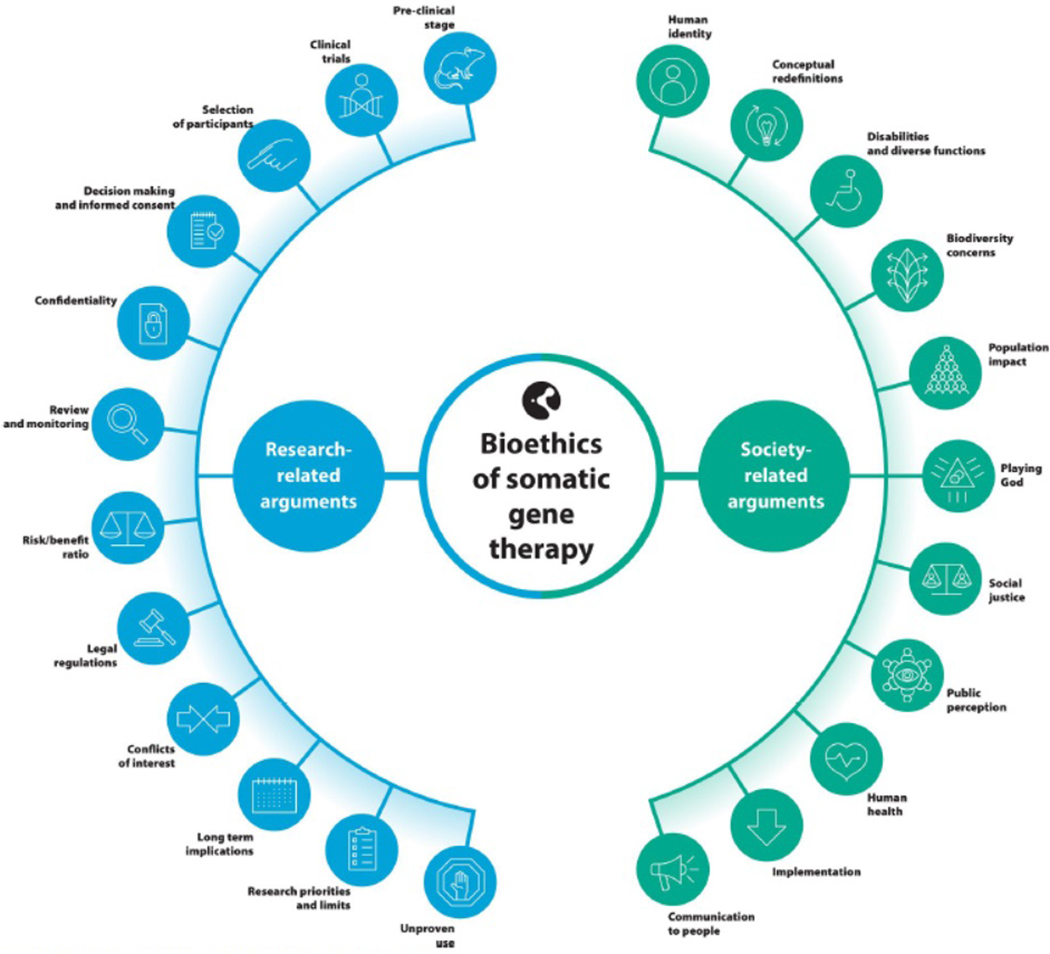
Categories grouped in research-related and society-related.

In total, 189 arguments were extracted from the included articles. These arguments were classified into 23 categories. All categories were grouped into two broad themes: research-related and society-related (Figure 2). We present all research-related and society-related arguments by category and with references in the Supplementary Material section (S7 Appendix). Below we describe some relevant features of the categories, with the reference number of the article listed in Table 1 where they are mentioned.

### Research-related categories

#### Pre-clinical stage

As many articles agreed on the need for animal testing to evaluate safety, efficacy, and long-term effects (31, 35, 52, 56, 57, 59, 71, 97, 99, 100, 117, 123, 124, 155, 189, 191, 214), others argued that even when this is important, it is not always possible to extrapolate directly from animal experiments to human studies (7, 10, 17, 18, 22, 64, 88, 154, 161, 189, 209). Not only is it necessary to test the gene therapy technology itself, but also basic pathophysiology studies are required because there is difficulty in establishing causality in the occurrence of the disease (10, 45, 110, 161, 184, 189).

#### Clinical trials

Although some arguments are around the idea that clinical trials on somatic gene therapy are new and could have high/uncertain risks (10, 18, 22, 28, 32, 40, 41, 68, 90, 102, 104, 114, 117, 174, 175, 121), an article stated that these trials are not fundamentally different from those associated with other experimental therapies (173). Regarding adverse events in trials, some articles argued that even if they are present, they should not invalidate the therapy itself, as it is experimental and many patients are seriously ill (38, 45, 124, 154, 174, 175). This could be related to the idea that there should not be a delay in starting clinical trials, because this could also be a harm to people suffering from diseases that somatic gene therapy could prevent or treat (31, 81, 97, 104, 113, 143, 211). However, one concern was that many clinical trials lack adequate statistical power to draw valid conclusions about possible racial or ethnic differences in response to or toxicities of new treatments (141). The need for public input in the research process is emphasized (5, 10, 16, 53, 62, 66, 81, 141, 148, 149, 150, 151, 152, 184, 210, 213).

#### Selection of participants

It is reported that there is a pressure to enroll record numbers of human subjects in record numbers of trials (207), but it is difficult to ensure fairness in the selection of subjects (7, 22, 31, 33, 40, 55, 64, 83, 97, 117, 129, 153, 176, 192, 200). Some argue that it could be justified in life-threatening diseases without any therapeutic alternative (55, 56, 57, 72, 74, 89, 100, 101, 110, 123, 158, 162, 183). On the contrary, end-stage disease should not be used to justify exposing participants to greater risks (196). But there is also a risk of exploitation related to what we call collateral affective benefits (hope and altruism) for research participants (196). It is reminded that the good of society should not come at the expense of individual persons (193, 200), and that society’s ethical commitments to people living today should be prioritized over those who may benefit in the future from gene therapy (176). It is claimed that it is unethical to recruit subjects from economically disadvantaged countries because they may not have access to gene therapy in the future, but, on the other hand, people from both developing and developed countries have something to gain by participating in gene therapy trials (214).

#### Decision making and informed consent

Regarding the decision-making process of potential participants in the trials, many arguments focus on informed consent itself. One of them highlights that consent form is an influential component of the consent process (209), another that informed consent seems to protect institutions and not participants (151) and some that there could be problems with understanding the nature of the intervention and risks for participants (1, 14, 42, 51, 104, 114, 149, 152, 165, 184, 201, 213). Several argued that participants may decide based on the hope that they will benefit themselves (28, 31, 32, 35, 104, 117, 130, 213) or that they will stop struggling with life-threatening diseases (51, 60, 66, 69). In summary, there are concerns based on evidence that the research subjects could overestimate the benefits and provide invalid informed consent (174, 176, 200, 204, 205, 209). It is important to provide very detailed information to patients participating in gene therapy trials to prevent unrealistic hopes (170, 196). The risks should be communicated even if they are unlikely to happen (8, 12, 18, 46, 50, 51, 159, 160, 214). It is also emphasized that receiving insufficient information about treatment is a main concern (144). On the other hand, some people prefer to wait for strong evidence before considering enrolling in a clinical trial (8, 73, 86). So far, we have mentioned empirical problems. In relation to conceptual problems that affect practice, many authors argue that the term gene therapy referring to research brings confusion and intensifies existing problems of informed consent (26, 31, 36, 40, 78, 174, 196, 201, 204, 205, 209). It should be clear that personal benefit does not overlap with the scientific purpose of the study (9, 13, 89, 95, 117, 122, 209) and that the benefits for the participants are not the same as the benefits for society (19, 174).

It is said that we should not only rely on the consent process to determine an acceptable level of harm, burden, or risk of harm (196) but also that informed consent could require a different strategy than usual to guarantee genuine decisions (51, 70, 81, 125, 138, 142, 148, 149, 189). Another thing to consider is that gene therapy could be irreversible, so the right to revoke one’s consent is not applicable here compared to continuing medical treatment and should be carefully explained (50).

#### Confidentiality

Many articles point out the difficulties in protecting privacy and confidentiality (4, 12, 36, 47, 64, 97, 162, 171, 187, 197, 198, 217), and that the information obtained during trials could be prejudicial to the individuals treated or to their families (50, 171, 187, 197, 198, 217).

#### Review and monitoring

As some articles discuss, there is no need for a special evaluation of the somatic gene therapy protocol (100, 107, 216) because somatic gene therapy arises ethical issues similar to other medical technologies/treatments (4, 6, 12, 18, 19, 22, 28, 32, 37, 38, 41, 44, 50, 63, 65, 66, 69, 70, 71, 72, 73, 76, 77, 78, 85, 93, 94, 96, 100, 102, 105, 111, 114, 122, 124, 126, 128, 143, 158, 162, 168, 171, 175, 178, 179, 181, 185, 191, 216). Others focus on the idea that there is a need for special evaluation and audit of somatic gene therapy protocols (11, 35, 40, 45, 57, 62, 81, 100, 113, 118, 121, 124, 131, 150, 154, 159, 160, 188, 190, 192, 200, 202, 210, 213), because gene therapy has very specific and unique ethical complexities compared to other medical practices (2, 39, 46, 71, 90, 119, 190, 208). Therefore, the bioethical implications of these experiments must be carefully considered (5, 16, 20) and security issues should not be confused with ethical issues (32). The protocol should be strictly followed, and any changes to the protocol must be documented (110, 115, 62, 89, 115, 137, 145, 187, 188) and should be an effective means of control and discipline after the protocol is approved (162). There is an obligation to avoid harm (19, 40, 87) and any adverse event must be reported (46, 62, 89, 115, 145).

The ethical complexity of gene therapy should not be approached only with an ethics committee (2, 147, 151, 154, 158, 159, 160, 162) and the public should be involved in the review and monitoring protocols as necessary (127).

#### Risk/benefit ratio

There is a claim that gene therapy should be treated as a conventional medical therapy when determining risk/benefit ratios (192) because the risks do not appear to be different from those encountered by any standard medical therapy (85). But other articles reveal that gene therapy has novel properties that can affect humans in unpredictable ways (7, 16, 61, 63, 64, 70, 90). Probabilities and outcomes for adverse events related to gene transfer are difficult to define (7, 10, 18, 22, 40, 42, 51, 63, 67, 104, 114, 117, 165, 184, 190). Gene therapy raises concerns about long-term safety and efficacy (12, 16, 17, 31, 40, 41, 45, 59, 60, 61, 63, 64, 67, 69, 76, 77, 89, 90, 105, 123, 166, 175, 182) and about serious and/or irreversible side effects (10, 17, 18, 23, 43, 50, 54, 60, 64, 69, 71, 85, 86, 88, 90, 100, 101, 114, 126, 167, 176, 183, 192).

Principal risks include technical issues in terms of the quality and stability of transgene expression (17, 31, 41, 59, 70, 85, 90, 110, 161, 168, 183, 184, 192, 196, 200, 202, 213), transfer of an unwanted gene, administration of replication-competent virus or bacterial contamination of vector preparation (177, 196, 202), immune response against both the vector and the transgene (54, 62, 118, 161, 164, 165, 168, 169, 175, 176, 177, 194, 196, 202, 213), activation of oncogene or inactivate a tumor suppressor gene caused by gene vector (164) and unintentional modification of germinal cells (31, 54, 64, 67, 85, 88, 107, 114, 117, 125, 126, 164, 175, 177, 180, 202).

On the other hand, viral vectors seem effective but are still not quite safe (17, 39, 61, 62, 64, 70, 71, 73, 90, 99, 100, 110, 118, 131, 142, 161, 165, 167, 183, 187, 196, 202, 213), non-viral vectors could be safer but still not efficient (17, 39, 62, 67, 70, 73, 131) and transgene expression worked but in long term is limited (142).

There are difficulties in balancing benefits and risks in relation to the burden and prognosis of the disease (18, 34, 40, 41, 48, 63, 95, 100, 104, 114, 121, 125, 190), but also because the risks are uncertain and cannot be reduced to a single utility (176, 193).

Furthermore, difficulties in the balance of risk/benefit relate to how potential social benefits should be balanced against individual risks (196, 201). There could be subtle social benefits of gene therapy (88, 100, 125). The problem with social benefit is that it can be as broad or narrow as one chooses (201). Beneficence is based on the potential for net benefit in the entire population while doing minimal harm to the individual (32, 81), and the distinction between medical benefits and collateral benefits is highlighted (196).

#### Conflicts of interest

The difficulties in managing conflicts of interest were highlighted in several articles (33, 39, 40, 53, 77, 85, 93, 100, 102, 121, 124, 115, 145, 146, 188, 207, 213), showing that important stakeholders have deep interests in gene therapy as a product (127, 155). Therefore, due to the great investments, scientists face a high pressure for success to develop gene therapy (4, 53, 117, 121). It is clear that clinical investigators should not have a personal financial relationship with companies that may benefit from the results (46). It is also shared that conflicts of interest do not need to be financial. They can be personal. For example, most Institutional Review Boards members in medical schools are employees of those institutions and have personal relationships with researchers (207). The overlapping roles could lead to potential conflicts in subject recruitment (104).

#### Regulations

Some articles expressed that the regulatory system is likely to be challenged by gene therapy (6, 21, 22, 31, 45, 67, 66, 68, 69, 121, 159, 160, 190). Regulations cannot be a general “blanket,” but each type of gene therapy must be evaluated on its own merits and risk analysis (149). However, others showed that gene therapy research is, without any scientific or medical basis, the most highly regulated procedure in medicine (135). Gene therapy is subject to too strict rules and is affected by overregulation (65, 68). No other form of therapy has ever been subjected to such strict control in its development and clinical trials as somatic gene therapy (179). Therefore, overregulation of gene therapy can lead to increased bureaucracy (207) and can profoundly slow its testing and ultimate adoption (135). An article suggests a worldwide accepted and controlled bioethics convention for somatic gene therapy (126).

#### Research priorities and limits

Some articles proposed that gene therapy per se is no longer being debated, but its application to particular diseases or particular patients is (179, 193, 216). In this sense, some authors mention that gene therapy used in diseases should be evaluated in advance (71, 85, 101, 125) or that the goal of the therapy has yet to be determined (175). There is also a back and forth about when to apply gene therapy. One position is that there should be more efforts to prevent rather than treat (4). The other is that gene therapy should not be a “first line” of defense therapy as long as an alternative is available (18). About priorities, there is a concern about who should decide what to investigate: companies, scientists or other? Pharmaceutical companies and other corporate interests often determine research priorities, which may not be aligned with public health needs (4, 191). Furthermore, scientists should decide about gene therapy research priorities on the basis of enlightened and broad-based public opinion (156).

The need to redefine the rights and responsibilities of all involved actors is noted (14, 17, 109, 117, 150, 152, 155, 184, 210, 213), as well as the need for public participation in genetic research policy (200). Lay people and stakeholders should be involved in the ethics discussion about gene therapy (4, 53, 58) as human gene pools are viewed as collective property. Public debate is necessary (50). But, with so many stakeholders, it could be difficult to design a regulation considering both political and cultural differences (17, 62, 60, 63, 64, 68, 76, 83, 85, 120, 127, 152, 201).

#### Unproven use

Unproven use refers to pre-approval, non-trial access to potentially beneficial therapies (3). For some rare diseases, experimental therapies such as gene therapy may be the only way to provide a treatment option (3). Patients who have exhausted other therapeutic options may not meet the restrictive criteria for inclusion in the trial (3). However, a failed use attempt with gene therapy may make a patient unable to try similar intervention again (215). In this sense, companies that might produce gene therapies want to “preserve the pool of future customers” and the reputation image, so they restrict unproven use (215). Moreover, since some gene therapies are one-dose treatments and the rare diseases patients are a small number of customers, there could be a commercial disincentive for unproven use (215).

#### Long term implications

The need to consider long-term implications was raised in some articles (4, 154, 162, 164) along with the need for adequate follow-up and ongoing care for the participants (10, 22, 54). However, this is not easy, as several factors seem to complicate the achievement of follow-up of patients participating in gene therapy trials (187).

### Society-related categories

#### Human identity

Those who do not believe that somatic gene therapy could change human identity state that the essence of the human person is not something that we can change at will, regardless of our technological capabilities (216). Human identity is more than a pool of genes (127) and is constantly redefined in biomedicine (76, 91, 105). Furthermore, an article states that gene therapy objectifies the disease in the person rather than the person (217).

However, others declare that somatic gene therapy could modify human identity, humanness or personal perception (11, 19, 27, 47, 69, 79, 101, 103, 109, 123, 131, 133, 191, 199, 212, 216) and could threaten human dignity (208). The body could be perceived as an enemy or as a source of weakness that is perfectible by technology (133), and eventually, the use of gene therapy could make certain human individuals cease to exist (4, 103). Gene therapy could reshape the ideas on how to live better (2), that effort is part of what makes us appreciate our life, so we do not have to eliminate all the pain or suffering (47). If we do so, we could lose our caring characteristics (47). Finally, gene therapy is said to not be used to change human traits (162).

#### Conceptual redefinitions

Gene therapy could open up some conceptual redefinitions. Some authors announce that could create a need for new disease/illness prevention and treatment concepts (11, 14, 49, 81, 110, 113, 122, 126, 133, 208). Additionally, it could be difficult to distinguish enhancement from treatment (11, 14, 29, 44, 47, 64, 66, 72, 74, 80, 81, 85, 94, 96, 97, 101, 102, 109, 110, 113, 114, 120, 122, 126, 132, 179, 185), and enhancement or eugenic therapy could be captured as human genetic therapy (167). In this sense, experiments in somatic gene therapy cannot be tainted by past associations with eugenics (172). Biotechnology is said to highlight moral problems, but not create them (44). Another conceptual issue that appears in some articles is that there are no ethical differences between germline and somatic gene therapy (25, 29) and that we are not conceptually forced to allow all types of gene therapy once we allow one (96).

#### Disability and diverse functions

Gene therapy could have an impact on social attitudes toward disability (133). On the one hand, gene therapy could not increase discrimination, but could make us aware of it (6, 81). On the other hand, the possibility of treatments could lead to more discrimination for disabled people (47). This is because diverse functions or bodies do not imply disabilities to prevent or treat, for example, deafness, but that community may argue that the only reason that deafness confers any disadvantages in society is because of societal discrimination (47). Also, in some cases, disability could be an integrated aspect of a person’s identity (133). Some articles mention that it is not necessary to overcome every human “limitation” (4, 47, 79, 81, 83, 91, 103, 105), and instead of working on solutions based on social bias, we need to think again about our social values (47).

#### Biodiversity concerns

There seems to be little concern about the impact of gene therapy on biodiversity (4). Gene therapy could replace the use of animal tissue culture used in current treatments (164), but the manufacture of gene therapy could be hazardous to the environment (1). In another sense, this field seems to avoid the issue that we are part of the environment because we put an anthropocentric distance ourselves from nature as if it were something different from human beings (4), and so gene therapy needs to consider the environmental effects on genes (4, 47, 49, 50).

#### Population impact

Gene therapy could have an impact on the population in different ways. To start, gene therapy research is a significant step in science evolution and therefore for well-being of humanity (40, 65, 67, 69, 70, 72, 74, 76, 78, 83, 105, 106, 107, 118, 124, 126). However new approaches have novel properties that may affect humans in unpredictable ways (142). There is a need to consider broad and long-range research consequences: public health, environmental and evolutionary concerns (200, 201).

Gene therapy for one person could have adverse repercussions on others (16, 27, 37, 44, 70, 77, 82, 85, 90, 93, 97, 114, 121, 126, 157, 200), for example, by making genetic diseases more prevalent in each generation after somatic gene therapy (37, 43, 202). In this sense, it is said that it could modify human evolution (37, 43, 76, 77, 81, 82, 91, 93, 94, 96, 101, 109, 122, 123, 126, 157, 167, 183, 184, 212, 217) because “bad” genes are needed from the viewpoint of the species (106). In opposition, other article advised that gene therapy will not affect human evolution (165).

Gene therapy could increase the possibility of the development of other new genetic technologies that have undesirable consequences (4, 35, 71, 72, 80, 93, 94, 96, 97, 101, 106, 122, 123, 128, 165, 183, 191, 199). For example, this could lead us to accept eugenic medical goals (4, 49, 52, 74, 81, 85, 94, 96, 157, 172, 208, 217), to a willingness to modify the color of the skin or change personality (167, 171) or that we are logically committed to accepting germline therapy (44, 72, 122, 208).

Despite the fact that gene therapy is offered with a focus on individual patient choice (70, 72, 79), it could motivate/-deepen conflicts between values (17, 35, 101, 107, 121, 152, 163) and turn social problems into genetic problems (4, 29, 85, 93). In addition, gene therapy could raise issues of fairness, justice, or equity in access to therapy (69, 67, 75, 81). Gene therapy could cause population aging (180) and longevity could cause loneliness and overpopulation, despite improving quality of life (1).

#### Social justice

Across social justice and similarly to what happened to other biomedical innovations, gene therapy could only be available in countries or for people with high income (1, 14, 17, 21, 33, 34, 36, 76, 77, 79, 90, 96, 101, 102, 183, 189, 197). It could be discriminatory to people who do not have access to gene therapy (11, 28, 36, 63, 81, 84, 101, 123, 185, 198, 212). An article argued that these economic inequities could affect human biology (112). Some propose that justice debates should take seriously the fact of scarcity in the field of gene therapy (195, 197), because it may also relegate funding from other areas of healthcare (4, 21, 32, 34, 36, 38, 61, 64, 69, 83, 75, 77, 79, 85, 112, 119, 125, 197, 202). The fact that gene therapy could be cost-effective compared to current therapies (50, 53, 55, 69, 143, 162, 164, 189, 202, 215) opens the possibility that gene therapy can be available for universal access to health care (86, 197).

#### Public perception

There is an ambivalence about the perception of gene therapy (208). Some authors show that people are unaware of the term “gene therapy” and its availability (69, 86, 97, 126). Others reported that there is no public trust in gene therapy (4, 8, 127) and that gene therapy has a long way to go before gaining widespread acceptance (180). The frequent reasons for not accepting gene therapy are fears of adverse effects, high cost, and a belief that it went against nature (180, 216). There are concerns about the political uses of gene technology, genetic discrimination, and misuse of power (180, 208). The possible consequences of manipulating genes or designing humans arise fear (9, 15, 60, 86, 93, 97, 98, 101, 105, 106, 126, 212). People think it is a risky procedure (127). It still provokes negative emotional reactions due to the stories of deaths (23, 62, 121, 131, 150, 163, 165, 210). On the other hand, many articles describe that there is high public support for the use of gene therapy to cure serious diseases but not for human enhancement (9, 19, 45, 50, 61, 63, 66, 67, 73, 74, 81, 85, 90, 97, 101, 106, 107, 113, 144, 167, 180, 212). Gene therapy is seen by most as a desirable extension to the range of available medical options (179) and people are interested in learning about gene therapy (212). The guarantee of sound research in general and the safety of patients is crucial for public support and recruitment (146).

With regard to religions, if it is for therapeutic purposes, gene therapy is accepted and encouraged, as long as proper precautions are taken (186, 198) considering that genetic manipulation leads to a delicate issue about soul alteration (186).

#### Human health

A common argument with respect to human health is that gene therapy could prevent and/or treat serious diseases that cause humanity to suffer and improve quality of life (60, 64, 68, 69, 73, 74, 80, 83, 84, 133, 140, 143, 169, 182, 185, 192, 199, 211). Furthermore, it could be the only possibility of treatment in particular diseases (11, 23, 31, 43, 50, 60, 62, 68, 70, 110, 111, 123, 128, 175, 179, 181, 182, 183, 185, 192). Therefore, there is a moral obligation to develop gene therapy if we consider it to be the only treatment for particular diseases (12, 19, 33, 36, 76, 125, 129, 194). It is also underlined that gene therapy has many potential applications, in addition to its application in monogenetic diseases (59, 62, 64, 69, 70, 73, 145, 161, 175, 181). Not only what gene therapy could do, but how: gene therapy may provide a curative rather than a symptomatic approach to diseases (143), holds the promise of preventing diseases (155) and restoring functions (175). An article presents that the progress in gene therapy is clearly relevant to women’s health for understanding and treating common diseases (197). Two important points were that gene therapy could avoid anxiety associated with the life-threatening nature of the underlying disease (53) and that therapeutic abortion could be rare if genetic diseases could be treated (53, 129).

#### Implementation

Gene therapy could create problems in its implementation in medicine (38, 59, 66, 67, 68, 131, 159, 165, 193, 194, 213). Specific standard operational procedures and cooperation between healthcare workers may be needed (64). Furthermore, some authors said that a genetic diagnosis is needed prior to therapy, so it should already be available (56, 81, 84, 123, 189). Therefore, if alternative treatment exists, the use of gene therapy will depend on its efficiency, costs, and level of discomfort for patients (59).

#### Communication with society

Many articles support the need for public trust on the basis of proper knowledge and transparency in the research process (14, 15, 17, 62, 68, 66, 81, 84, 90, 100, 108, 150, 152, 161, 163, 165, 184, 213). Hence, public opinion should be adequately informed about gene therapy (81), and scientists must spend adequate time communicating science to the media (8, 137, 149, 212).

According to some authors, terminology has been shown to influence risk and benefit perception (205, 209), and here the term “gene therapy” used in research does not reflect whether it is a therapy or research (50, 53, 54, 89, 93, 95, 104, 107, 113, 117, 124, 150, 161, 201, 204, 213). It has been shown that the potential of somatic cell gene therapy may have been exaggerated, especially in relation to the timeline of its successful implementation (202, 216) with a tendency to amplify potential benefits and minimize potential risks (68, 66, 78, 124, 134, 190). Reinforcing this, the oversell of gene therapy research could cause a slowdown in gene therapy if something bad happens (155). As an emotionally volatile topic, if no patient is helped, the negative reaction can halt the entire field of gene therapy (169). However, advances have been made during the last few years, and there are reasons to hope clinically important results will be presented (175).

#### Playing God

Some articles came with the topic of “playing God,” referring to actions that could be done without any limit and have serious effects on people’s lives, as someone could have unlimited power. Some stated that humankind should not play God (76, 81, 91, 106, 122, 157, 167, 208), others that we are not playing God with gene therapy, as science is a human activity (127), and that there may be both proper and improper ways of “playing God” (203).

## Discussion

To our knowledge, this article constitutes the first systematic review of reasons in bioethics for somatic gene therapy. Systematic reviews of reasons are relatively new in descriptive ethics. Recent articles that have applied this method include bioethical debates about organoids models^20^, permissibility in research with great apes^21^, germline modifications^22^, genome editing in non-human animals^23^, among others. Systematic reviews of reasons provide broader perspective of the chosen topic.

Somatic gene therapy following conventional techniques has the potential to be a major step in science and humanity’s well-being^5^. After analyzing all the arguments provided in this review, we can agree that at the same time, this technology could have repercussions on a massive scale and we do not have clear answers how to deal with these challenges^6,11,24^.

The impact that gene therapy could have -or already has had- on society is different from any other social impact of a non-genetic health-related biotechnology^13,24,25^. The role that we give to genes impacts in how we understand our health and the functionality of our body^26^. We are targeting genes as mediators of human illness, which play a role in some kinds of disease; but they are not always the whole explanation and the social considerations surrounding them should be seriously considered^13,27^. Somatic gene therapy on a massive scale could have repercussions on human identity^24^. For example, many deaf people do not consider themselves as a person with disability, but rather identify deafness as personal feature that is part of their identity^25^. If somatic gene therapy could play a role in “treating” this diverse function through genes, then diverse functions could be seen just as a genetics problem. In this context, people with deafness might be seen as people with a genetic abnormality that may have impact on the identity of those who do not consider themselves with an abnormality. And in this context, deciding not to “treat the abnormality” will be out of a personal decision, but on the social framework of an abnormal who actually needs to correct the abnormality. The deaf situation is one example of how somatic gene therapy is very close to the genetic determination idea, and this is one of the reasons it is not similar to other non-genetics biotechnologies. Another specific issue is that we cannot guarantee that all people could eventually access this kind of therapy. This should be considered in advance, because there is a great risk of transforming genetics modifications into a social disadvantage based on the economic situation of a person^25^.

Although new techniques in the genetic field, like CRISPR, raise ethical challenges and attention, we want to highlight the problems of conventional somatic gene therapy that already exist^26^. Debates on certain topics should not be marginalized because other challenges appear, but rather that there is a minimum consensus on the discussion^24,28^, which has not yet been consolidated in the case of conventional somatic gene therapy. As we demonstrate in this review of arguments, procedural, conceptual, and social issues about somatic gene therapy remain issues that need to be addressed.

Moreover, society should be part of the debate, defining priorities and limits in gene therapy research, ethical permissibility and nuances regarding its acceptance by certain communities and for certain uses^9,24^. All of this could also have positive influences on the development of the somatic gene therapy field^9^.

Our analysis should be interpreted in light of the following limitations. First, there were some terms that were not included in the search strategy that may be associated with ethics and bioethics, for example informed consent or risks/benefits. This was intentional to make the systematic review feasible. Second, we are aware that a different group of researchers could have selected or grouped the included reasons in a different way. Third, we did not assess the scientific validity of the articles included.

## Conclusion

This article is a starting point in a systematic re-evaluation of the ethical arguments before somatic gene therapy will transform into a massive-scale procedure. Our study provides a database of existing challenges and arguments of somatic gene therapy and may serve as the basis of normative analysis.

## Supplementary Material

Bioethics what Supp 2

Bioethics what Supp 1

Bioethics what Supp 3

Bioethics what Supp 4

Bioethics what Supp 5

Bioethics what Supp 7

Bioethics what Supp 6

## Figures and Tables

**Table 1.** ID, authors and year of articles included in the cohort.

1= Traulsen et al. 2008	74= McKenny et al. 1999	147= Kimmelman 2003
2= Addison et al. 2017	75= Farrelly 2004	148= Pattee 2008
3= Thrasher et al. 2013	76= Cole-Turner 1997	149= Delhove et al. 2020
4= Barns et al. 2000	77= Fost 1993	150= Horst 2007
5= Carmen 2001	78= Churchill et al. 1998	151= Zallen 1996
6= Hughes 2019	79= Chadwick et al. 1998	152= Sato et al. 2006
7= Riva et al. 2019	80= Friedmann 2019	153= Kimmelman 2008
8= Steele 2000	81= Gustafson 1992	154= Kimmelman 2005
9= Bonatti et al. 2002	82= Lacadena 2005	155= Anderson 1991
10= Ledley 1995	83= Williams 2002	156= Areen 1985
11= Holtug 1997	84= Kaplan et al. 2000	157= Leavitt 2001
12= Baird 1994	85= Gage 1987	158= Black 1998
13= Kim et al. 2009	86= Costea et al. 2009	159= Cornetta et al. 2002
14= Podhajcer et al. 1998	87= Savulescu 2001	160= Cornetta 2003
15= Sturgis et al. 2005	88= Editorial 1993	161= Orkin et al. 1995
16= Kimmelman 2012	89= Health Department of the United Kingdom Gene Therapy Advisory Committee 2001	162= Committee 1992
17= Freire et al. 2014	90= Wirth et al. 2013	163= Priest 2009
18= Swazo 2006	91= Messer 1999	164= Ragni 2002
19= Walter 2003	92= McGleenan 1995	165= Temin 1990
20= Fischer 2000	93= Larson 1990	166= Lagay 1999
21 = Pepper et al. 2018	94= Launis 2002	167= Lebo et al. 1991
22= Ledley 1991	95= Carmen 1993	168= Weatherall 1995
23= Friedmann 2004	96= Holtug 1993	169= Anderson 1990
24= Lowenstein 2008	97= Walters 1991	170= Nelles et al. 2015
25= Moseley 1991	98= Krimsky 1990	171= Motulsky 1989
26= King et al. 2005	99= Anderson 1985	172= Ledley 1987
27= Campbell et al. 1998	100= Leiden 2000	173= Ledley 1992
28= Tauer 1990	101= Anderson 1989	174= Kimmelman 2007
29= Scully 2001	102= Patel 1993	175= Lyngstadaas 2002
30= Kimmelman et al. 2005	103= Ellliot 1993	176= Kimmelman 2008
31 = King et al. 2008	104= Kahn 2008	177= Glass et al. 1999
32= Nicholson et al. 1995	105= Zänker et al. 1997	178= Norfolk et al. 1990
33= Levin 2016	106= Macer et al. 1995	179= Bayertz et al. 1994
34= Flotte 2015	107= Richter et al. 1998	180= Xiang et al. 2015
35= Fletcher 1985	108= Editorial 1996	181 = Risco 2006
36= Penticuff 1994	109= Fitzgerald 2002	182= Espin-Villacres et al. 2001
37= Shannon 1999	110= Ruiz-Perez 1993	183= Rodriguez Yunta 2003
38= Fost 1992	111= Casanova Perdomo 2011	184= Agudelo Veléz et al. 2013
39= Bernstein et al. 2004	112= Green 2005	185= Smith et al. 2010
40= Zhang 2008	113= Dickens 1996	186= Pace 2004
41= Haan 1990	114= Areen 1990	187= Ledley et al. 1992
42= Kimmelman 2012	115= Wilson 2009	188= Wilson 2010
43= Valenzuela 2003	116= Robin et al. 1987	189= Walters 1986
44= Fletcher 1990	117= Palmer 1991	190= Kimmelman 2008
45= Nevin 1998	118= Nunes et al. 1996	191 = Dyer 1997
46= Kaji et al. 2001	119= Neel 1997	192= McDonough 1997
47= Goering 2000	120= Barreiro 1999	193= Bunch et al. 2000
48= Drugan et al. 1987	121 = Baramt 2001	194= Friedmann 1990
49= Bertolaso et al. 2010	122= Crisp 1995	195= Farrelly 2004
50= Royal Commission on New Reproductive	123= Gafo 2000	196= Nycum et al. 2007
Technologies 1994		
51 = Kaspar et al. 2009	124= Friedmann 2000	197= Fletcher 1998
52= Danks 1993	125= Swiss Academy of Medical Sciences 1999	198= Kraj 2002
53= Dimichele et al. 2003	126= Winter et al. 1995	199= Sadler et al. 2004
54= Giangrande 2004	127= Bruce 2006	200= Juengst 1990
55= Dimichele 2005	128= Stahl 2015	201 = Kong 2004
56= Friedmann et al. 1972	129= Fletcher 1983	202= Karpati et al. 1997
57= Anderson et al. 1980	130= Turriff et al. 2019	203= Walter 1999
58= Hoshino 1995	131= Lenk et al. 2007	204= Henderson et al. 2006
59= Weatherall 1991	132= Ebbesen et al. 2006	205= Kimmelman et al. 2005
60= Ashcroft 2004	133= Scully et al. 2004	206= Kimmelman 2009
61= Robinson et al. 1996	134= Benjaminy et al. 2014	207= Gilbert 2008
62= Wolf et al. 2009	135= Miller 1995	208= Kass 2000
63= Spink et al. 2004	136= Cohen-Haguenauer 1995	209= Henderson et al. 2004
64= Roth et al. 2002	137= Steele 2000	210= Teichler Zallen 2000
65= Mavilio 2010	138= Brooks et al. 2019	211= Anderson 1992
66= Rabino 2003	139= Aiyegbusi et al. 2020	212= Robillard et al. 2013
67= Jin et al. 2008	140= Konduros 2019	213= Stockdale 1999
68= Cohen-Haguenauer 1997	141= King et al. 2010	214= Ponder et al. 2008
69= Hillman et al. 1996	142= Dettweiler et al. 2001	215= Chapman et al. 2019
70= Smith 2003	143= Gansbacher 2002	216= Porter 1990
71= Hoose 1990	144= Robillard et al. 2014	217= Keenan 1990
72= Fuchs 2006	145= Górecki 2001	
73 Amor 2001	146= Shalala 2000	

*Note:* For full cohort details, see S5 Appendix.
