# Supplementary material for: Bioethics of somatic gene therapy: what do we know so far?
Source: Curr Med Res Opin. Author manuscript; Available in PMC 2025 Jan 30. (PMC11780552; doi:10.1080/03007995.2023.2257600)
Supplement: Bioethics what Supp 2 [file NIHMS2040356-supplement-Bioethics_what_Supp_2.docx]

Appendix 2: Search strategy in all databases.

| *Database* | *Search strategy* |
| --- | --- |
| PubMed | ("Genetic Therapy"[Mesh] OR "Gene Transfer Techniques"[Mesh]) AND ("Ethics"[Mesh] OR "Bioethics"[Mesh] OR "Morals"[Mesh] OR "Social Validity, Research"[Mesh] OR "Patient Acceptance of Health Care"[Mesh] OR "Value of Life"[Mesh] OR "ethics" [Subheading])  Filter used on Species: Humans  Filter used on Languages: English and Spanish |
| Lilacs | "Bioetica" or "Etica" or “Moral” [Descriptor de asunto] and "Terapia Genetica" [Descriptor de asunto] or "Tecnicas de Transferencia de Genes" [Descriptor de asunto] |
| PhilPapers | "ethics" AND "gene" AND (transfer \| therapy) |
| Google Scholar | Spanish search: (Etica OR Bioetica) AND (Terapia genetica OR Terapia genica OR transferencia genetica)  English search: (Ethics OR Bioethics OR Ethical) AND ("Gene therapy" OR "Gene transfer") AND Research  Filter used: Patents or citations not included |
