# Supplementary material for: Bioethics of somatic gene therapy: what do we know so far?
Source: Curr Med Res Opin. Author manuscript; Available in PMC 2025 Jan 30. (PMC11780552; doi:10.1080/03007995.2023.2257600)
Supplement: Bioethics what Supp 1 [file NIHMS2040356-supplement-Bioethics_what_Supp_1.pdf]

**Title**	Bioethics of human gene transfer: a systematic review of reasons
**Authors**	Paola Buedo, Katarzyna Klas, Marcin Waligora.
**Introduction**	
Rationale	Human gene transfer is a method that introduces new genetic material to human cells which aim to treat or to improve a health condition (John Hopkins Medicine, 2005).<br>Many preclinical and clinical research evaluate the therapeutic potential of human gene transfer (Alhakamy et al, 2021). However, the extensive research efforts are accompanied by unresolved ethical discussions (Kimmelman, 2008).<br>Identifying and classifying systematically the ethical challenges and moral implications of this novel medical technology is part of science's responsibility. The results could guide research and future application of this technology and so enables the field to invest time and resources in the right way.
Objective	To identify and classified reasons for and against gene transfer that have been documented in the scientific literature.
**Methods**	
Type of systematic review	When we discuss an ethical question, it is important to find out which reasons have been given for and against a certain topic and how they have been used (Strech & Sofaer, 2012). We will conduct a systematic review of reasons to identify, classify and present the reasons given in the scientific literature (Strech & Sofaer, 2012).
Eligibility criteria	Publications will be eligible if they contain an explicit reason for or against gene transfer. 'Reasons' may refer to acceptability, importance, value, morality, ethics or bioethics. 'Human gene transfer' should focus on somatic cells and have therapeutical goals.<br><u>Inclusion criteria</u><br>- Normative articles focusing on somatic gene transfer/therapy and its ethical/bioethical aspects.<br>- Articles focusing on public perception or use of somatic gene transfer/therapy.<br>- Articles focusing on professional's and researchers about ethical aspects of somatic gene transfer/therapy.<br>- Narrative reviews, editorials, commentaries, opinions, letters, guidelines, and policy recommendations.<br>- Articles in English or Spanish.<br><u>Exclusion criteria</u><br>- Articles that focused entirely on the ethics/bioethics of germline gene transfer or genome editing.<br>- Articles that focused on intrauterine, fetal or prenatal gene transfer.<br>- Reports of interventional studies of gene transfer/therapy.<br>- Articles from press.<br>- Books, books chapters, comments on books, and congress abstract/posters.

Information sources	We will conduct the search in PubMed, Lilacs, PhilPapers and Google Scholar databases. We selected these databases because they cover a comprehensive area of biomedical and philosophical publications from all over the world. We add the most important Latin-American database (Lilacs) because we want to be sensitive to cultural or otherwise region-dependent differences.<br>We will perform the search without time restrictions.<br>We will download the data to Excel and/or EndNote.
Search strategy	PubMed<br>("Genetic Therapy"[Mesh] OR "Gene Transfer Techniques"[Mesh]) AND ("Ethics"[Mesh] OR "Bioethics"[Mesh] OR "Morals"[Mesh] OR "Social Validity, Research"[Mesh] OR "Patient Acceptance of Health Care"[Mesh] OR "Value of Life"[Mesh] OR "ethics" [Subheading])<br><u>Filter use on Species</u>: Humans<br><u>Filter use on Languages</u>: English and Spanish<br><br>Lilacs<br>"Bioetica" or "Etica" or "Moral" [Descriptor de asunto] and "Terapia Genetica" [Descriptor de asunto] or "Tecnicas de Transferencia de Genes" [Descriptor de asunto]<br><br>PhilPapers<br>"ethics" AND "gene" AND (transfer | therapy)<br><br>Google Scholar<br>- (Etica OR Bioetica) AND (Terapia genetica OR Terapia genica OR transferencia genetica)<br>- (Ethics OR Bioethics OR Ethical) AND ("Gene therapy" OR "Gene transfer") AND Research<br><u>Filter use</u>: Patents or citations not included
Data selection and screening	Once we carried out the searches in each database, we will move the outcomes to Endnote and/or Excel. Duplicates will be removed. Two reviewers will screen titles and abstracts independently applying inclusion and exclusion criteria. Discrepancies will be discussed and agreed. If needed, third person, an arbiter, will be involved.<br>After title/abstract screening, we will obtain full texts, stored, and screened. Two researchers will discuss the eligible papers. In case of disagreement over inclusion or exclusion of a paper, a third person, an arbiter, will be involved.
Data collection process	Once the cohort of studies is defined, we will make a spreadsheet with descriptive information of all the publications: ID number, title, author/s, year of publication; language; publication type; publication field; journal, quotation.
Data analyses and synthesis	We will use the constant comparative method (CCM) (Gibbs, 2008). We will extract arguments and reasons contained in each publication (verbatim where possible). Each researcher will organise these

	arguments and reasons in a second spreadsheet with: the ID number of publication, arguments, and the broad and narrow reason/s. Researchers will compare the proposed reasons and discuss them following the CCM method.<br>At the end of the process we will summarize the reasons and arguments in qualitative and quantitative way.
Outcomes	Outcomes will be report following PRISMA recommendations when possible (Page et al, 2021).
Risk of bias	The method of a systematic review of reasons is not amenable to formal analysis of risk of bias.
Availability of data	Protocol, results of the pilot and raw data will be shared on the Open Science Framework.
**References**	

- Alhakamy N, Curiel D & Berkland C. The era of gene therapy: from preclinical development to clinical application. Drug Discov Today. 2021;27:S1359-6446(21)00157-4. Epub ahead of print.
- Gibbs G. Analysing qualitative data. Qualitative Research Kit. London: SAGE; 2008.
- John Hopkins Medicine. Gene Transfer Research. Baltimore: John Hopkins Medicine; 2005. Available from: https://www.hopkinsmedicine.org/institutional_review_board/guidelines_policies/guidelines/gene_transfer.html
- Kimmelman J. The ethics of human gene transfer. Nat Rev Genet. 2008;9:239–244.
- Page M, McKenzie J, Bossuyt P, Boutron I, Hoffmann T, Mulrow C et al. The PRISMA 2020 statement: an updated guideline for reporting systematic reviews. BMJ. 2021;372:n71
- Strech D & Sofaer N. How to write a systematic review of reasons. J Med Ethics. 2012;38:121e126.
