# Supplementary material for: Bioethics of somatic gene therapy: what do we know so far?
Source: Curr Med Res Opin. Author manuscript; Available in PMC 2025 Jan 30. (PMC11780552; doi:10.1080/03007995.2023.2257600)
Supplement: Bioethics what Supp 3 [file NIHMS2040356-supplement-Bioethics_what_Supp_3.docx]

Appendix 3: Data extraction documents design (originally in Excel forms)

Data extraction document 1 (for article characteristics)

| **Article Title** | **Article Type** | **Journal Title** | **Year** | **Language** | **Field** | **Authors** | **Database** | **Reference Number** |
| --- | --- | --- | --- | --- | --- | --- | --- | --- |

Data extraction document 2 (for article characteristics)

| **Article** | **Author** | **Affiliation** | **Country** |
| --- | --- | --- | --- |

Data extraction document 3 (for arguments extraction)

| **Category** | **Argument** | **Reference Number** |
| --- | --- | --- |
