# Supplementary material for: Bioethics of somatic gene therapy: what do we know so far?
Source: Curr Med Res Opin. Author manuscript; Available in PMC 2025 Jan 30. (PMC11780552; doi:10.1080/03007995.2023.2257600)
Supplement: Bioethics what Supp 4 [file NIHMS2040356-supplement-Bioethics_what_Supp_4.docx]

Appendix 4: The PRISMA-Ethics Reporting Guideline.

Manuscript: **Bioethics of somatic gene therapy: what do we know so far?**

From:

Kahrass H, et al. PRISMA-Ethics - Reporting guideline for systematic reviews on ethics literature: development, explanations and examples. 10.31219/osf.io/g5kfb [Preprint]. Available from <https://osf.io/g5kfb>

| **No. of item** | | **Reported in # section (including paragraphs)** |
| --- | --- | --- |
| **Title** | | |
| 1 | **Title**: Identify the report as a systematic review. | Not reported |
| **Abstract** | | |
| 2 | **Structured summary**: Provide a structured summary including, as applicable: objectives; ethics literature eligibility criteria; information sources; ethics literature appraisal and synthesis methods; included publications; synthesis of results; limitations of evidence; interpretation (conclusions and implications of key findings); funding; systematic review registration number. | Reported in a modified way with respect to journal requests for unstructured abstracts. |
| **Introduction** | | |
| 3 | **Rationale**: Describe the rationale for the review in the context of existing knowledge. | Introduction (Paragraphs 1-4) |
| 4 | **Objectives:** Provide an explicit statement of the objective(s) or question(s) the review addresses. | Introduction (Paragraph 5) |
| **Methods** | | |
| 5 | **Eligibility criteria**: Specify the inclusion and exclusion criteria for the review (e.g., years considered, language, type of publication) and give a rationale. | Methods (Eligibility criteria) |
| 6 | **Search strategy:** Specify all databases, registers, websites, organisations, reference lists and other sources searched or consulted to identify publications. Specify the date when each source was last searched or consulted. Provide the rationale for using the information sources and present the full search strategy, including any limits and filters used, such that it could be repeated. | Methods (Search strategy);  S2 Appendix |
| 7 | **Selection process**: Specify the methods used to decide whether a publication met the inclusion criteria of the review, including how many reviewers screened each record and each publication retrieved, whether they worked independently and how disagreements were resolved, and if applicable, details of automation tools used in the process. | Methods (Selection process) |
| 8 | **Data extraction**: Indicate which sections of the publication were analysed and how were the data extracted from the publication. If applicable, state the software and details of automation tools used in the process. | Methods (Data extraction);  S3 Appendix |
| 9 | **Identification of codes and themes:** Explain the process of assigning the codes, themes, or items (e.g. inductive, deductive, a combination of deductive and inductive strategies), if applicable. If so, describe the process for coding of data (e.g. line by line coding to search for concepts), including how many reviewers analysed each publication. List and define all other variables for which information were sought (e.g. participant and intervention characteristics, funding sources). Describe any assumptions made about any missing or unclear information. | Methods (Identification of codes and themes) |
| 10 | **Quality appraisal:** Indicate whether a quality appraisal was performed and why, and if yes, outline the quality appraisal process and its results (e.g. how many reviewers assessed each study, did they work independently). | Methods (Quality appraisal) |
| 11 | **Synthesis methodology:** Identify the synthesis methodology or theoretical framework which underpins the synthesis, and describe the rationale for choice of methodology (e.g. thematic analysis, content analysis, critical interpretive synthesis, grounded theory synthesis, narrative synthesis). Describe any methods used to tabulate or visually display results of individual studies and syntheses. | Methods (Data extraction) |
| **Results** | | |
| 12 | **Publication selection process**: Describe the results of the search and selection process, from the number of publications identified in the search to the number of studies included in the review, with a flow diagram (including reasons for exclusions at each stage). | Results (Publication selection process);  Figure 1 |
| 13 | **Characteristics of publications**: For each publication, included in the review, present characteristics for which data were extracted and provide the citations. | Results (Characteristics of publications);  S5 Appendix;  S6 Appendix |
| 14 | **Results of syntheses:** Present the results (e.g. new systematization of issues or arguments) and reference publications as evidence. | Results (Results of syntheses);  S7 Appendix |
| 15 | **Quotations**: Provide original wording to illustrate themes, if applicable. | Not applicable |
| **Discussion** | | |
| 16 | **Summary**: Summarize the main findings and provide a general interpretation of the results in the context of other evidence; consider their relevance to key groups (e.g. health care workers, academics, other decision maker). | Discussion (Paragraphs 2-5) |
| 17 | **Strength and limitations**: Discuss strengths and limitations of the publications included in the review and the review process itself. | Discussion (Paragraph 7) |
| 18 | **Conclusions**: Discuss implications of the results for practice, policy and/or future research. | Discussion (Paragraph 6) |
| **Other Information** | | |
| 19 | **Registration and protoco**l: State whether the review was registered, or state that the review was not registered. If yes, provide registration information for the review, including register name and registration number. Indicate where the review protocol can be accessed, or state that a protocol was not prepared. Describe and explain any amendments to information provided at registration or in the protocol. | Methods (Paragraph 1);  S1 Appendix |
| 20 | **Support:** Describe sources of funding for the systematic review and other support (e.g., supply of data); role of funders for the systematic review. | Funding |
| 21 | **Competing interests:** Declare any competing interests of review authors. | Disclosure statement |
| 22 | **Availability of data, code and other materials:** Report which of the following are publicly available and where they can be found: template information collection forms; information extracted from included studies; information used for all analyses; analytic code; any other materials used in the review. | **-** |
