# Supplementary material for: Bioethics of somatic gene therapy: what do we know so far?
Source: Curr Med Res Opin. Author manuscript; Available in PMC 2025 Jan 30. (PMC11780552; doi:10.1080/03007995.2023.2257600)
Supplement: Bioethics what Supp 5 [file NIHMS2040356-supplement-Bioethics_what_Supp_5.docx]

Appendix 5: Details of the articles included in the systematic review.

| **ID** | **Authors** | **Title** | **Year** | **Journal** | **Database** | **Language** | **Field** |
| --- | --- | --- | --- | --- | --- | --- | --- |
| 1 | Traulsen JM, Bjornsdóttir I, Almarsdóttir AB | 'I'm Happy if I Can Help'. Public views on future medicines and gene-based therapy in Iceland | 2008 | Community Genetics | PubMed | English | Genetics |
| 2 | Addison C, Lassen J | "My whole life is ethics!" Ordinary ethics and gene therapy clinical trials | 2017 | Medical Anthropology | PubMed | English | Social Sciences |
| 3 | Gaspar HB, Swift S, Thrasher AJ | "Special exemptions": should they be put on trial? | 2013 | Molecular Therapy | PubMed | English | Biotechnology |
| 4 | Barns I, Schibeci R, Davison A, Shaw R | "What do you think about genetic medicine?" Facilitating sociable public discourse on developments in the new genetics | 2000 | Science, Technology, & Human Values | PubMed | English | Science |
| 5 | Carmen IH | A death in the laboratory: the politics of the Gelsinger aftermath | 2001 | Molecular Therapy | PubMed | English | Biotechnology |
| 6 | Hughes JJ | A defense of limited regulation of human genetic therapies | 2019 | Cambridge Quarterly of Healthcare Ethics | PubMed | English | Bioethics |
| 7 | Riva L, Petrini C | A few ethical issues in translational research for gene and cell therapy | 2019 | Journal of Translational Medicine | PubMed | English | Medicine |
| 8 | Steele FR | A matter of trust | 2000 | Molecular Therapy | PubMed | English | Biotechnology |
| 9 | Bonatti J, Haeusler C, Klaus A, Fink M, Hammerer-Lercher A, Laufer G | Acceptance of gene therapy by the heart surgery patient | 2002 | European Journal of Cardio-thoracic Surgery | PubMed | English | Surgery |
| 10 | Ledley FD | After gene therapy: issues in long-term clinical follow-up and care | 1995 | Advances in Genetics | PubMed | English | Genetics |
| 11 | Holtug N | Altering humans — The case for against human gene therapy | 1997 | Cambridge Quarterly of Healthcare Ethics | PhilPapers | English | Bioethics |
| 12 | Baird PA | Altering humans genes: social, ethical, and legal implications | 1994 | Perspectives in Biology and Medicine | PubMed | English | Medicine |
| 13 | Kim SY, Schrock L, Wilson RM, Frank SA, Holloway RG, Kieburtz K, de Vries RG | An approach to evaluating the therapeutic misconception | 2009 | IRB: Ethics and Human Research | PubMed | English | Bioethics |
| 14 | Podhajcer O, Pitossi F, Boyesen McReddie C | Aspectos eticos de la terapia genica | 1998 | Medicina | LILACS | Spanish | Medicine |
| 15 | Sturgis P, Cooper H, Fife-Schaw C | Attitudes to biotechnology: estimating the opinions of a better-informed public | 2005 | New Genetics and Society | PubMed | English | Genetics |
| 16 | Kimmelman J | Beyond human subjects: risk, ethics, and clinical development of nanomedicine | 2012 | Journal of Law, Medicine & Ethics | PubMed | English | Bioethics |
| 17 | Freire JE, Medeiros SC, Lopes Neto AV, Monteiro Júnior JE, Sousa AJ, Rocha AJ, Menezes LM | Bioethical conflicts of gene therapy: a brief critical review | 2014 | Revista da Associação Médica Brasileira | LILACS | English | Medicine |
| 18 | Swazo NK | Calculating Risk/Benefit in X-Linked Severe combined immune deficiency disorder (X-SCID) gene therapy trials: the task of ethical evaluation | 2006 | Journal of Medicine and Philosophy | PubMed | English | Bioethics |
| 19 | Walter JJ | Catholic reflections on the human genome | 2003 | The National Catholic Bioethics Quarterly | PubMed | English | Bioethics |
| 20 | Fischer A | Cautious advance. Gene therapy is more complex than anticipated | 2000 | EMBO Reports | PubMed | English | Biology |
| 21 | Pepper MS, Alessandrini M, Pope A, Van Staden W, Green RJ | Cell and gene therapies at the forefront of innovative medical care: implications for South Africa | 2018 | South African Medical Journal | PubMed | English | Medicine |
| 22 | Ledley FD | Clinical considerations in the design of protocols for somatic gene therapy | 1991 | Human Gene Therapy | PubMed | English | Genetics |
| 23 | Friedmann T | Clinical gene therapy: lessons from the ether dome | 2004 | Molecular Therapy | PubMed | English | Biotechnology |
| 24 | Lowenstein PR | Clinical trials in gene therapy: ethics of informed consent and the future of experimental medicine | 2008 | Current Opinion in Molecular Therapeutics | PubMed | English | Biotechnology |
| 25 | Moseley R | Commentary: maintaining the somatic/germ-line distinction: some ethical drawbacks | 1991 | Journal of Medicine and Philosophy | PubMed | English | Bioethics |
| 26 | King NM, Henderson GE, Churchill LR, Davis AM, Hull SC, Nelson DK, Parham-Vetter PC, Rothschild BB, Easter MM, Wilfond BS | Consent forms and the therapeutic misconception: the example of gene transfer research | 2005 | IRB: Ethics and Human Research | PubMed | English | Bioethics |
| 27 | Campbell A, Glass KC, Charland LC | Describing our "humanness": can genetic science alter what it means to be "human"? | 1998 | Science and Engineering Ethics | PhilPapers | English | Bioethics |
| 28 | Tauer CA | Does human gene therapy raise new ethical questions? | 1990 | Human Gene Therapy | PubMed | English | Genetics |
| 29 | Scully JL | Drawing a line: situating moral boundaries in genetic medicine | 2001 | Bioethics | PubMed | English | Bioethics |
| 30 | Kimmelman J, Levenstadt A | Elements of style: consent form language and the therapeutic misconception in phase 1 gene transfer trials | 2005 | Human Gene Therapy | PubMed | English | Genetics |
| 31 | King N, Cohen-Haguenauer O | En route to ethical recommendations for gene transfer clinical trials | 2008 | Molecular Therapy | PubMed | English | Biotechnology |
| 32 | Nicholson S, Pandha HS, Harris JD, Waxman J | Ethical and regulatory issues in gene therapy | 1995 | British Journal of Urology | PubMed | English | Urology |
| 33 | Levin AV | Ethical considerations in gene therapy | 2016 | Ophthalmic Genetics | PubMed | English | Ophthalmology |
| 34 | Flotte TR | Ethical implications of the cost of molecularly targeted therapies | 2015 | Human Gene Therapy | PubMed | English | Genetics |
| 35 | Fletcher JC | Ethical issues in and beyond prospective clinical trials of human gene therapy | 1985 | Journal of Medicine and Philosophy | PhilPapers | English | Bioethics |
| 36 | Penticuff J | Ethical issues in genetic therapy | 1994 | Journal of Obstetric, Gynecologic & Neonatal Nursing | PubMed | English | Nursing |
| 37 | Shannon TA | Ethical issues in genetics | 1999 | Theological Studies | PubMed | English | Theology |
| 38 | Fost N | Ethical issues in genetics | 1992 | Pediatric Clinics of North America | PubMed | English | Pediatrics |
| 39 | Bernstein M, Bampoe J, Daar AS | Ethical issues in molecular medicine of relevance to surgeons | 2004 | Canadian Journal of Surgery | PubMed | English | Surgery |
| 40 | Zhang X | Ethical reflection on human gene therapy in the Chinese context | 2008 | Journal International de Bioéthique | PubMed | English | Bioethics |
| 41 | Haan EA | Ethics and the new genetics | 1990 | Journal of Paediatrics and Child Health | PubMed | English | Pediatrics |
| 42 | Kimmelman J | Ethics, ambiguity aversion, and the review of complex translational clinical trials | 2012 | Bioethics | PubMed | English | Bioethics |
| 43 | Valenzuela CY | Etica cientifica de la terapia genica de individuos. Urgencia de la cirugia genica del ADN | 2003 | Revista Medica de Chile | LILACS | Spanish | Medicine |
| 44 | Fletcher JC | Evolution of ethical debate about human gene therapy | 1990 | Human Gene Therapy | PubMed | English | Genetics |
| 45 | Nevin NC | Experience of gene therapy in the United Kingdom | 1998 | Annals of the New York Academy of Sciences | PubMed | English | Science |
| 46 | Kaji EH, Leiden JM | Gene and stem cell therapies | 2001 | JAMA | PubMed | English | Medicine |
| 47 | Goering S | Gene therapies and the pursuit of a better human | 2000 | Cambridge Quarterly of Healthcare Ethics | PubMed | English | Bioethics |
| 48 | Drugan A, Müler O, Evans M | Gene therapy | 1987 | Fetal Therapy | PubMed | English | Obstetrics |
| 49 | Bertolaso M, Olsson J, Picardi A, Rakela J | Gene therapy and enhancement for diabetes (and other diseases): the multiplicity of considerations | 2010 | Diabetes/Metabolism Research and Reviews | PubMed | English | Endocrinology |
| 50 | Royal Commission on New Reproductive Technologies | Gene therapy and genetic alteration | 1994 | Human Gene Therapy | PubMed | English | Genetics |
| 51 | Kaspar RW, Wills CE, Kaspar BK | Gene therapy and informed consent decision making: nursing research directions | 2009 | Biological Research for Nursing | PubMed | English | Nursing |
| 52 | Danks DM | Gene therapy and related novel forms of treatment | 1993 | The Medical Journal of Australia | PubMed | English | Medicine |
| 53 | Dimichele D, Miller FG, Fins JJ | Gene therapy ethics and haemophilia: an inevitable therapeutic future? | 2003 | Haemophilia | PubMed | English | Hematology |
| 54 | Giangrande PLF | Gene therapy for hemophilia? No | 2004 | Journal of Thrombosis and Haemostasis | PubMed | English | Hematology |
| 55 | Dimichele D | Gene therapy for hemophilia? The debate reframed | 2005 | Journal of Thrombosis and Haemostasis | PubMed | English | Hematology |
| 56 | Friedmann T, Roblin R | Gene therapy for human genetic disease? | 1972 | Science | GS English | English | Science |
| 57 | Anderson WF, Fletcher JC | Gene therapy in human beings: when is it ethical to begin? | 1980 | The New England Journal of Medicine | GS English | English | Medicine |
| 58 | Hoshino K | Gene therapy in Japan: current trends | 1995 | Cambridge Quarterly of Healthcare Ethics | PubMed | English | Bioethics |
| 59 | Weatherall DJ | Gene therapy in perspective | 1991 | Nature | PubMed | English | Science |
| 60 | Ashcroft RE | Gene therapy in the clinic: whose risks? | 2004 | Trends in Biotechnology | PubMed | English | Biotechnology |
| 61 | Robinson KD, Abernathy E, Conrad KJ | Gene therapy of cancer | 1996 | Seminars in Oncology Nursing | PubMed | English | Nursing |
| 62 | Wolf SM, Gupta R, Kohlhepp P | Gene therapy oversight: lessons for nanobiotechnology | 2009 | Journal of Law, Medicine & Ethics | PhilPapers | English | Bioethics |
| 63 | Spink J, Geddes D | Gene therapy progress and prospects: bringing gene therapy into medical practice: the evolution of international ethics and the regulatory environment | 2004 | Gene Therapy | PubMed | English | Genetics |
| 64 | Roth RI, Fleischer NM | Gene therapy: applications to pharmacy practice | 2002 | Journal of the American Pharmaceutical Association | PubMed | English | Pharmacy |
| 65 | Mavilio F | Gene therapy: back on track? | 2010 | EMBO Reports | PubMed | English | Biology |
| 66 | Rabino I | Gene therapy: ethical issues | 2003 | Theoretical Medicine | PubMed | English | Medicine |
| 67 | Jin X, Yang YD, Li YM | Gene therapy: Regulations, ethics and its practicalities in liver disease | 2008 | World Journal of Gastroenterology | PubMed | English | Gastroenterology |
| 68 | Cohen-Haguenauer O | Gene therapy: regulatory issues and international approaches to regulation | 1997 | Current Opinion in Biotechnology | PubMed | English | Biotechnology |
| 69 | Hillman AL, Brenner MK, Caplan AL, Carey J, Champey Y, Culver KW, Drummond MF, Freund DA, Holmes EW, Kelley WN, Kolata G, Levine MN, Levy E, Schondelmeyer SW, Velu T, Wilson JM. | Gene therapy: socioeconomic and ethical issues. A roundtable discussion | 1996 | Human Gene Therapy | PubMed | English | Genetics |
| 70 | Smith KR | Gene therapy: theoretical and bioethical concepts | 2003 | Archives of Medical Research | PubMed | English | Medicine |
| 71 | Hoose B | Gene therapy: where to draw the line | 1990 | Human Gene Therapy | PubMed | English | Genetics |
| 72 | Fuchs M | Gene therapy. An ethical profile of a new medical territory | 2006 | The Journal of Gene Medicine | PubMed | English | Medicine |
| 73 | Amor D | Gene therapy. Principles and potential applications | 2001 | Australian Family Physician | PubMed | English | Medicine |
| 74 | McKenny GP, Aguilar-Cordova E | Gene transfer for therapy or enhancement | 1999 | Human Gene Therapy | PubMed | English | Genetics |
| 75 | Farrelly C | Genes and equality | 2004 | Journal of Medical Ethics | PubMed | English | Bioethics |
| 76 | Cole-Turner R | Genes, religion and society: the developing views of the churches | 1997 | Science and Engineering Ethics | PubMed | English | Bioethics |
| 77 | Fost N | Genetic diagnosis and treatment. Ethical considerations | 1993 | The American Journal of Diseases of Children | PubMed | English | Pediatrics |
| 78 | Churchill LR, Collins ML, King NM, Pemberton SG, Wailoo KA | Genetic research as therapy: implications of "gene therapy" for informed consent | 1998 | Journal of Law, Medicine & Ethics | PubMed | English | Bioethics |
| 79 | Chadwick R, Levitt M | Genetic technology: A threat to deafness | 1998 | Medicine, Health Care and Philosophy | PubMed | English | Medicine |
| 80 | Friedmann T | Genetic therapies, human genetic enhancement, and ... eugenics? | 2019 | Gene Therapy | PubMed | English | Genetics |
| 81 | Gustafson JM | Genetic therapy: ethical and religious reflections | 1992 | Journal of Contemporary Health Law and Policy | PubMed | English | Law |
| 82 | Ramón JR | Genetica y bioetica: lo posible y lo deseable | 2005 | Revista de la Academia Canaria de Ciencias | GS Spanish | Spanish | Science |
| 83 | Williams ED | Genetics and bioethics: the current state of affairs | 2002 | Revista Latinoamericana de Bioética | PubMed | English | Bioethics |
| 84 | Kaplan JC, Junien C | Genomics and medicine: an anticipation. From Boolean Mendelian genetics to multifactorial molecular medicine | 2000 | Comptes rendus de l'Academie des Sciences | PubMed | English | Science |
| 85 | Gage JL | Government regulation of human gene therapy | 1987 | Jurimetrics Journal | PubMed | English | Law |
| 86 | Costea I, Isasi R, Knoppers BM, Lillicrap D | Haemophilia gene therapy: the patients-perspective | 2009 | Haemophilia | PubMed | English | Hematology |
| 87 | Savulescu J | Harm, ethics committees and the gene therapy death | 2001 | Journal of Medical Ethics | PubMed | English | Bioethics |
| 88 | Editorial | Hasty compassion | 1993 | The Lancet | PubMed | English | Medicine |
| 89 | Health Department of the United Kingdom Gene Therapy Advisory Committee | Guidance on making proposals to conduct gene therapy research on human subjects | 2001 | Human Gene Therapy | PubMed | English | Genetics |
| 90 | Wirth T, Parker N, Ylä-Herttuala S | History of gene therapy | 2013 | Gene | PubMed | English | Genetics |
| 91 | Messer N | Human cloning and genetic manipulation: some theological and ethical issues | 1999 | Studies in Christian Ethics | PubMed | English | Bioethics |
| 92 | McGleenan T | Human gene therapy and slippery slope arguments | 1995 | Journal of Medical Ethics | PubMed | English | Bioethics |
| 93 | Larson EJ | Human gene therapy and the law: an introduction to the literature | 1990 | Emory Law Journal | PubMed | English | Law |
| 94 | Launis V | Human gene therapy and the slippery slope argument | 2002 | Medicine, Health Care and Philosophy | PubMed | English | Medicine |
| 95 | Carmen IH | Human gene therapy: a biopolitical overview and analysis | 1993 | Human Gene Therapy | PubMed | English | Genetics |
| 96 | Holtug N | Human gene therapy: down the slippery slope? | 1993 | Bioethics | PubMed | English | Bioethics |
| 97 | Walters L | Human gene therapy: ethics and public policy | 1991 | Human Gene Therapy | PubMed | English | Genetics |
| 98 | Krimsky S | Human gene therapy: must we know where to stop before we start? | 1990 | Human Gene Therapy | PubMed | English | Genetics |
| 99 | Anderson WF | Human gene therapy: scientific and ethical considerations | 1985 | Journal of Medicine and Philosophy | GS English | English | Bioethics |
| 100 | Leiden JM | Human gene therapy: the good, the bad, and the ugly | 2000 | Circulation Research | PubMed | English | Hematology |
| 101 | Anderson WF | Human gene therapy: why draw a line? | 1989 | Journal of Medicine and Philosophy | PubMed | English | Bioethics |
| 102 | Patel PI | Identification of disease genes and somatic gene therapy: an overview and prospects for the aged | 1993 | Journal of Gerontology | PubMed | English | Gerontology |
| 103 | Ellliot R | Identity and the ethics of gene therapy | 1993 | Bioethics | PubMed | English | Bioethics |
| 104 | Kahn J | Informed consent in human gene transfer clinical trials | 2008 | Human Gene Therapy | PubMed | English | Genetics |
| 105 | Zänker KS, Huber HP | Interdisciplinary forum: from genetic diagnosis to gene therapy in oncology | 1997 | Journal of Cancer Research and Clinical Oncology | PubMed | English | Oncology |
| 106 | Macer DR, Akiyama S, Alora AT, Asada Y, Azariah J, Azariah H, Boost MV, Chatwachirawong P, Kato Y, Kaushik V, Leavitt FJ, Macer NY, Ong CC, Srinives P, Tsuzuki M | International perceptions and approval of gene therapy | 1995 | Human Gene Therapy | PubMed | English | Genetics |
| 107 | Richter G, Bacchetta MD | Interventions in the human genome: some moral and ethical considerations | 1998 | Journal of Medicine and Philosophy | PubMed | English | Bioethics |
| 108 | Editorial | Keeping faith in gene manipulation | 1996 | Nature | PubMed | English | Science |
| 109 | Fitzgerald KT | Knowledge without wisdom: human genetic engineering without religious insight | 2002 | Christian Bioethics | PubMed | English | Bioethics |
| 110 | Ruiz-Perez G | La terapia genetica: observaciones para una perspectiva etica | 1993 | Scripta Theologica | GS Spanish | Spanish | Theology |
| 111 | Casanova Perdomo AR | Las tecnologias de manipulacion de genes humanos como imperativo tecnologico: analisis desde la optica del principalismo bioetico y el principio de responsabilidad | 2011 | Revista Latinoamericana de Bioética | GS Spanish | Spanish | Bioethics |
| 112 | Green RM | Last word: imagining the future | 2005 | Kennedy Institute of Ethics Journal | PubMed | English | Bioethics |
| 113 | Dickens BM | Legal and ethical challenges in gene therapy | 1996 | Transfusion Science | PubMed | English | Hematology |
| 114 | Areen J, King P | Legal regulation of human gene therapy | 1990 | Human Gene Therapy | PubMed | English | Genetics |
| 115 | Wilson JM | Lessons learned from the gene therapy trial for ornithine transcarbamylase deficiency | 2009 | Molecular Genetics and Metabolism | GS English | English | Genetics |
| 116 | Robin SS, Markle GE, Curd M, Duster T, Lappé M, Mazur A | Let no one split asunder: controversy in human genetic engineering | 1987 | Politics and the Life Sciences | PubMed | English | Politics |
| 117 | Palmer JG | Liability considerations presented by human gene therapy | 1991 | Human Gene Therapy | PubMed | English | Genetics |
| 118 | Nunes FA, Raper SE | Liver-directed gene therapy | 1996 | Medical Clinics of North America | PubMed | English | Medicine |
| 119 | Neel JV | Looking ahead: some genetic issues of the future | 1997 | Perspectives in Biology and Medicine | PubMed | English | Medicine |
| 120 | Barreiro AJ | Los delitos relativos a la manipulacion genetica en sentido estricto | 1999 | Anuario de derecho penal y ciencias penales | GS Spanish | Spanish | Law |
| 121 | Baramt M | Making clinical trials safer for human subjects | 2001 | American Journal of Law & Medicine | PubMed | English | Law |
| 122 | Crisp R | Making the world a better place: genes and ethics | 1995 | Science and Engineering Ethics | PubMed | English | Bioethics |
| 123 | Gafo J | Manipulacion genetica | 2000 | Almogaren. Revista del Centro Teológico de Las Palmas | GS Spanish | Spanish | Theology |
| 124 | Friedmann T | Medical ethics. Principles for human gene therapy studies | 2000 | Science | PubMed | English | Science |
| 125 | Swiss Academy of Medical Sciences | Medical-ethical guidelines for somatic gene therapy in humans | 1999 | Schweiz Med Wochenschr | PubMed | English | Medicine |
| 126 | Winter SF, Roger HD | Medical, ethical and legal aspects of somatic gene therapy | 1995 | European Journal of Health Law | PubMed | English | Law |
| 127 | Bruce DM | Moral and ethical issues in gene therapy | 2006 | Human Reproduction & Genetic Ethics | PubMed | English | Bioethics |
| 128 | Stahl D | Moral evaluations of genetic technologies. The need for catholic social doctrine | 2015 | The National Catholic Bioethics Quarterly | PhilPapers | English | Bioethics |
| 129 | Fletcher JC | Moral problems and ethical issues in prospective human gene therapy | 1983 | Virginia Law Review | PubMed | English | Law |
| 130 | Turriff A, Blain D, Similuk M, Biesecker B, Wiley H, Cukras C, Sieving PA | Motivations and decision making processes of Men with X-linked retinoschisis considering participation in an ocular gene therapy trial | 2019 | American Journal of Ophthalmology | PubMed | English | Ophthalmology |
| 131 | Lenk C, Biller-Andorno N | Nanomedicine–emerging or re-emerging ethical issues? A discussion of four ethical themes | 2007 | Medicine, Health Care and Philosophy | PhilPapers | English | Medicine |
| 132 | Ebbesen M, Jensen TG | Nanomedicine: techniques, potentials, and ethical implications | 2006 | Journal of Biomedicine and Biotechnology | GS English | English | Biotechnology |
| 133 | Scully JL, Rippberger C, Rehmann-Sutter C | Non-professionals' evaluations of gene therapy ethics | 2004 | Social Science & Medicine | PubMed | English | Social Sciences |
| 134 | Benjaminy S, Bubela T | Ocular gene transfer in the spotlight: implications of newspaper content for clinical communications | 2014 | BMC Medical Ethics | PubMed | English | Bioethics |
| 135 | Miller HI | Overregulation is an unnecessary hindrance to human gene therapy | 1995 | Human Gene Therapy | PubMed | English | Genetics |
| 136 | Cohen-Haguenauer O | Overview of regulation of gene therapy in Europe: a current statement including reference to US regulation | 1995 | Human Gene Therapy | PubMed | English | Genetics |
| 137 | Steele F | Painful lessons | 2000 | Molecular Therapy | PubMed | English | Biotechnology |
| 138 | Brooks SP, Benjaminy S, Bubela T | Participant perspectives on a phase I/II ocular gene therapy trial | 2019 | Ophthalmic Genetics | PubMed | English | Ophthalmology |
| 139 | Aiyegbusi OL, Macpherson K, Elston L, Myles S, Washington J, Sungum N, Briggs M, Newsome PN, Calvert MJ | Patient and public perspectives on cell and gene therapies: a systematic review | 2020 | Nature | PubMed | English | Science |
| 140 | Konduros J | Patient testimonial: my experience on a gene therapy trial | 2019 | Transfusion and Apheresis Science | PubMed | English | Hematology |
| 141 | King WD, Wyatt GE, Liu H, Williams JK, DiNardo AD, Mitsuyasu RT | Pilot assessment of HIV gene therapy-hematopoietic stem cell clinical trial acceptability among minority patients and their advisors | 2010 | Journal of the National Medical Association | PubMed | English | Medicine |
| 142 | Dettweiler U, Simon P | Points to consider for ethics committees in human gene therapy trials | 2001 | Bioethics | PubMed | English | Bioethics |
| 143 | Gansbacher B | Policy statement on the social, ethical and public awareness issues in gene therapy | 2002 | The Journal of Gene Medicine | PubMed | English | Medicine |
| 144 | Robillard JM, Roskams-Edris D, Kuzeljevic B, Illes J | Prevailing public perceptions of the ethics of gene therapy | 2014 | Human Gene Therapy | PubMed | English | Genetics |
| 145 | Górecki DC | Prospects and problems of gene therapy: an update | 2001 | Expert Opinion on Emerging Drugs | GS English | English | Pharmacy |
| 146 | Shalala D | Protecting research subjects--what must be done | 2000 | The New England Journal of Medicine | PubMed | English | Medicine |
| 147 | Kimmelman J | Protection at the cutting edge: the case for central review of human gene transfer research | 2003 | Canadian Medical Association Journal | PubMed | English | Medicine |
| 148 | Pattee SR | Protections for participants in gene therapy trials: a patient's perspective | 2008 | Human Gene Therapy | PubMed | English | Genetics |
| 149 | Delhove J, Osenk I, Prichard I, Donnelley M | Public acceptability of gene therapy and gene editing for human use: a systematic review | 2020 | Human Gene Therapy | PubMed | English | Genetics |
| 150 | Horst M | Public expectations of gene therapy: scientific futures and their performative effects on scientific citizenship | 2007 | Science, Technology, & Human Values | GS English | English | Science |
| 151 | Zallen DT | Public oversight is necessary if human gene therapy is to progress | 1996 | Human Gene Therapy | PubMed | English | Genetics |
| 152 | Sato H, Akabayashi A, Kai I | Public, experts, and acceptance of advanced medical technologies: the case of organ transplant and gene therapy in Japan | 2006 | Health Care Analysis | PhilPapers | English | Health Care Sciences |
| 153 | Kimmelman J | Putting the shoe on the wrong foot: a reply to Ponder and Srivastava | 2008 | Haemophilia | PubMed | English | Hematology |
| 154 | Kimmelman J | Recent developments in gene transfer: risk and ethics | 2005 | BMJ | PubMed | English | Medicine |
| 155 | Anderson FW | Reflections: of hope and of concern | 1991 | Human Gene Therapy | PubMed | English | Genetics |
| 156 | Areen J | Regulating human gene therapy | 1985 | West Virginia Law Review | PubMed | English | Law |
| 157 | Leavitt WJ | Regulating human gene therapy: legislative overreaction to human subject protection failures | 2001 | Administrative Law Review | PubMed | English | Law |
| 158 | Black J | Regulation as facilitation: negotiating the genetic revolution | 1998 | The Modern Law Review | PubMed | English | Law |
| 159 | Cornetta K, Smith FO | Regulatory issues for clinical gene therapy trials | 2002 | Human Gene Therapy | PubMed | English | Genetics |
| 160 | Cornetta K | Regulatory issues in human gene therapy | 2003 | Blood Cells, Molecules and Diseases | PubMed | English | Hematology |
| 161 | Orkin SH, Motulsky AG | Report and recommendations of the panel to assess the NIH investment in research on gene therapy | 1995 | National Institutes of Health (not journal) | GS English | English | Science |
| 162 | Committee | Report of the Committee on the Ethics of Gene Therapy | 1992 | Human Gene Therapy | PubMed | English | Genetics |
| 163 | Priest SH | Risk communication for nanobiotechnology: to whom, about what, and why? | 2009 | Journal of Law, Medicine & Ethics | PhilPapers | English | Bioethics |
| 164 | Ragni MV | Safe passage: a plea for safety in hemophilia gene therapy | 2002 | Molecular Therapy | PubMed | English | Biotechnology |
| 165 | Temin HM | Safety considerations in somatic gene therapy of human disease with retrovirus vectors | 1990 | Human Gene Therapy | GS English | English | Genetics |
| 166 | Lagay FL | Science, rhetoric, and public discourse in genetic research | 1999 | Cambridge Quarterly of Healthcare Ethics | PubMed | English | Bioethics |
| 167 | Lebo RV, Golbus MS | Scientific and ethical considerations in human gene therapy | 1991 | Bailliere's Clinical Obstetrics and Gynaecology | PubMed | English | Obstetrics |
| 168 | Weatherall DJ | Scope and limitations of gene therapy | 1995 | British Medical Bulletin | PubMed | English | Medicine |
| 169 | Anderson WF | September 14, 1990: the beginning | 1990 | Human Gene Therapy | PubMed | English | Genetics |
| 170 | Nelles M, Stieger K, Preising MN, Kruse J, Lorenz B | Shared decision-making, control preferences and psychological well-being in patients with RPE65 deficiency awaiting experimental gene therapy | 2015 | Ophthalmic Research | PubMed | English | Ophthalmology |
| 171 | Motulsky AG | Societal problems in human and medical genetics | 1989 | Genome | PubMed | English | Genetics |
| 172 | Ledley FD | Somatic gene therapy for human disease: a problem of eugenics? | 1987 | Trends in Genetics | PubMed | English | Genetics |
| 173 | Ledley FD | Somatic gene therapy in gastroenterology: approaches and applications | 1992 | Journal of Pediatric Gastroenterology and Nutrition | PubMed | English | Gastroenterology |
| 174 | Kimmelman J | Stable ethics: enrolling non-treatment-refractory volunteers in novel gene transfer trials | 2007 | Molecular Therapy | PubMed | English | Biotechnology |
| 175 | Lyngstadaas A | Status and potential of gene therapy in clinical medicine. Assessment of an emerging health technology through systematic survey of clinical gene therapy protocols and published results | 2002 | International Journal of Technology Assessment in Health Care | PubMed | English | Health Care Sciences |
| 176 | Kimmelman J | Staunch protections: the ethics of haemophilia gene transfer research | 2008 | Haemophilia | PubMed | English | Hematology |
| 177 | Glass KC, Weijer C, Cournoyer D, Lemmens T, Palmour RM, Shapiro SH, Freedman B | Structuring the review of human genetics protocols, part III: gene therapy studies | 1999 | IRB: Ethics and Human Research | PubMed | English | Bioethics |
| 178 | Norfolk, Gallagher JC, Jessiman I | Submission to the Committee on the Ethics of Gene Therapy | 1990 | The Linacre Quarterly | PubMed | English | Theology |
| 179 | Bayertz K, Paslack R, Schmidt KW | Summary of "gene transfer into human somatic cells. State of the technology, medical risks, social and ethical problems: a report" | 1994 | Human Gene Therapy | PubMed | English | Genetics |
| 180 | Xiang L, Xiao L, Gou Z, Li M, Zhang W, Wang H, Feng P | Survey of attitudes and ethical concerns related to gene therapy among medical students and postgraduates in China | 2015 | Human Gene Therapy | PubMed | English | Genetics |
| 181 | Risco DL | Terapia genica e investigacion con celulas madre en la legislacion española | 2006 | Derecho y Salud | GS Spanish | Spanish | Law |
| 182 | Espin-Villacres V, Andrade-Vera K, Espin-Mayorga V | Terapia genica en medicina | 2001 | Boletín Médico del Hospital Infantil de México | LILACS | Spanish | Pediatrics |
| 183 | Rodriguez Yunta E | Terapia genica y principios eticos | 2003 | Acta Bioethica | LILACS | Spanish | Bioethics |
| 184 | Agudelo Vélez CA, Martínez Sánchez LM | Terapia génica: una opción de tratamiento y una controversia ética | 2013 | Salud Uninorte | GS Spanish | Spanish | Medicine |
| 185 | Smith RS, Piras BA, Smith CJ | The bioethics of gene therapy | 2010 | The National Catholic Bioethics Quarterly | PhilPapers | English | Bioethics |
| 186 | Pace A | The catholic theology of genetic manipulation | 2004 | The Linacre Quarterly | PubMed | English | Theology |
| 187 | Ledley FD, Brody B, Kozinetz CA, Mize SG | The challenge of follow-up for clinical trials of somatic gene therapy | 1992 | Human Gene Therapy | PubMed | English | Genetics |
| 188 | Wilson RF | The death of Jesse Gelsinger: new evidence of the influence of money and prestige in human research | 2010 | American Journal of Law & Medicine | PubMed | English | Law |
| 189 | Walters LR | The ethics of human gene therapy | 1986 | Nature | GS English | English | Science |
| 190 | Kimmelman J | The ethics of human gene transfer | 2008 | Nature | PubMed | English | Science |
| 191 | Dyer AR | The ethics of human genetic intervention: a postmodern perspective | 1997 | Experimental Neurology | PubMed | English | Neurosciences |
| 192 | McDonough PG | The ethics of somatic and germline gene therapy | 1997 | Annals of the New York Academy of Sciences | PubMed | English | Science |
| 193 | Bunch WH, Drennan JC | The ethics of the introduction of gene therapy into orthopaedic practice | 2000 | Clinical Orthopaedics and Related Research | PubMed | English | Orthopaedics |
| 194 | Friedmann T | The evolving concept of gene therapy | 1990 | Human Gene Therapy | PubMed | English | Genetics |
| 195 | Farrelly C | The genetic difference principle | 2004 | The American Journal of Bioethics | PubMed | English | Bioethics |
| 196 | Nycum G, Reid L | The harm-benefit tradeoff in “bad deal” trials | 2007 | Kennedy Institute of Ethics Journal | PubMed | English | Bioethics |
| 197 | Fletcher JC | The long view: how genetic discoveries will aid healthcare reform | 1998 | Journal of Women's Health | PubMed | English | Medicine |
| 198 | Kraj T | The magisterium and modern genetics | 2002 | The National Catholic Bioethics Quarterly | PubMed | English | Bioethics |
| 199 | Sadler TD, Zeidler DL | The morality of socioscientific issues: construal and resolution of genetic engineering dilemmas | 2004 | Science Education | GS English | English | Education |
| 200 | Juengst ET | The NIH "Points to Consider" and the limits of human gene therapy | 1990 | Human Gene Therapy | PubMed | English | Genetics |
| 201 | Kong WM | The regulation of gene therapy research in competent adult patients, today and tomorrow: implications of EU Directive 2001/20/EC | 2004 | Medical Law Review | PubMed | English | Law |
| 202 | Karpati G, Lochmüller H | The scope of gene therapy in humans: scientific, safety and ethical considerations | 1997 | Neuromuscular Disorders | PubMed | English | Neurosciences |
| 203 | Walter JJ | Theological issues in genetics | 1999 | Theological Studies | PubMed | English | Theology |
| 204 | Henderson GE, Easter MM, Zimmer C, King NM, Davis AM, Rothschild BB, Churchill LR, Wilfond BS, Nelson DK | Therapeutic misconception in early phase gene transfer trials | 2006 | Social Science & Medicine | PubMed | English | Social Sciences |
| 205 | Kimmelman J, Palmour N | Therapeutic optimism in the consent forms of phase 1 gene transfer trials: an empirical analysis | 2005 | Journal of Medical Ethics | PubMed | English | Bioethics |
| 206 | Kimmelman J | Tomorrow, interrupted? Risk, ethics, and medical advance in gene transfer | 2009 | Molecular Therapy | PubMed | English | Biotechnology |
| 207 | Gilbert S | Trials and tribulations | 2008 | Hastings Center Report | PubMed | English | Bioethics |
| 208 | Kass LR | Triumph or tragedy? The moral meaning of genetic technology | 2000 | The American Journal of Jurisprudence | PubMed | English | Law |
| 209 | Henderson GE, Davis AM, King NM, Easter MM, Zimmer CR, Rothschild BB, Wilfond BS, Nelson DK, Churchill LR | Uncertain benefit: investigators' views and communications in early phase gene transfer trials | 2004 | Molecular Therapy | PubMed | English | Biotechnology |
| 210 | Teichler Zallen D | US gene therapy in crisis | 2000 | Trends in Genetics | GS English | English | Genetics |
| 211 | Anderson WF | Uses and abuses of human gene transfer | 1992 | Human Gene Therapy | PubMed | English | Genetics |
| 212 | Robillard JM, Whiteley L, Johnson TW, Lim J, Wasserman WW, Illes J | Utilizing social media to study information-seeking and ethical issues in gene therapy | 2013 | Journal of Medical Internet Research | PubMed | English | Medicine |
| 213 | Stockdale A | Waiting for the cure: mapping the social relations of human gene therapy research | 1999 | Sociology of Health & Illness | GS English | English | Social Sciences |
| 214 | Ponder KP, Srivastava A | Walk a mile in the moccasins of people with haemophilia | 2008 | Haemophilia | PubMed | English | Hematology |
| 215 | Chapman CR, Moch KI, McFadyen A, Kearns L, Watson T, Furlong P, Bateman-House A | What compassionate use means for gene therapies | 2019 | Nature Biotechnology | PubMed | English | Biotechnology |
| 216 | Porter J | What is morally distinctive about genetic engineering? | 1990 | Human Gene Therapy | PubMed | English | Genetics |
| 217 | Keenan JF | What is morally new in genetic manipulation? | 1990 | Human Gene Therapy | PubMed | English | Genetics |
