# Supplementary material for: Bioethics of somatic gene therapy: what do we know so far?
Source: Curr Med Res Opin. Author manuscript; Available in PMC 2025 Jan 30. (PMC11780552; doi:10.1080/03007995.2023.2257600)
Supplement: Bioethics what Supp 7 [file NIHMS2040356-supplement-Bioethics_what_Supp_7.docx]

Appendix 7: Research-related arguments (Table 1) and society-related arguments (Table 2).

Table 1: Research-related arguments.

| **Category** | **Arguments** | **ID/s of article/s where the argument was/were extract** |
| --- | --- | --- |
| **Pre-clinical stage** | need for animal testing to evaluate safety, efficacy and long-term effects | 31, 35, 52, 56, 57, 59, 71, 97, 99, 100, 117, 123, 124, 155, 189, 191, 214 |
|  | it is not always possible to extrapolate directly from animal experiments to human studies | 7, 10, 17, 18, 22, 64, 88, 154, 161, 189, 209 |
|  | difficulty in establishing causality in disease occurrence and basic studies of pathophysiology are needed it | 10, 45, 110, 161, 184, 189 |
|  | genetic therapies should take into account environmental effects on genes | 4, 47, 49, 50 |
|  | | |
| **Clinical trials** | delay in initiating trials could be harmful to people who are suffering | 31, 81, 97, 104, 113, 143, 211 |
|  | adverse results do not invalidate gene therapy as is experimental | 38, 124, 154, 174 |
|  | there are no reports of major adverse reactions in the last gene therapy clinical trials | 45, 175 |
|  | need for public input in the research process | 5, 10, 16, 53, 62, 66, 81, 136, 139, 141, 148, 149, 150, 151, 152, 184, 210, 213 |
|  | gene therapy trials are new and could have high/uncertain risks | 10, 18, 22, 28, 32, 40, 41, 68, 90, 102, 104, 114, 117, 136, 174, 175, 121 |
|  | many gene therapy trials lack adequate statistical power to make valid conclusions about possible racial or ethnic differences | 141 |
|  | | |
| **Selection of participants** | participation in gene therapy trials can be beneficial for people both in developing and developed countries | 214 |
|  | could be justified in life-threatening diseases without any therapeutic alternative | 55, 56, 57, 72, 74, 89, 100, 101, 110, 123, 158, 162, 183 |
|  | genetic education can foster participant engagement | 166 |
|  | society's ethical commitments to people living today should be prioritized over those who may benefit in the future from gene therapy | 176 |
|  | the good of society should not come at the expense of individual persons | 193, 200 |
|  | there is a risk of exploitation related to what we call collateral affective benefits (hope and altruism) for research participants | 196 |
|  | the “terminally” of a participant situation should not be used to justify the “higher risk” than is permitted for a non-terminal participant | 196 |
|  | there is pressure to recruit a record number of human subjects to a record number of trials | 207 |
|  | it is unethical to recruit subjects from economically disadvantaged countries because they may not have access to gene therapy | 214 |
|  | difficult to ensure fairness in the selection of subjects | 7, 22, 31, 33, 40, 55, 64, 83, 97, 117, 129, 153, 176, 192, 200 |
|  | | |
| **Decision making and informed consent** | informed consent could require a different strategy than usual to guarantee genuine decisions | 51, 70, 81, 125, 138, 142, 148, 149, 189 |
|  | the consent form is an influential component to the consent process | 196, 209 |
|  | problems with understanding the nature of the intervention and risks for participants | 1, 14, 42, 51, 104, 114, 149, 152, 165, 184, 201, 213 |
|  | participants may decide based on the hope that they will benefit themselves | 28, 31, 32, 35, 51, 60, 66, 69, 104, 117, 130, 213 |
|  | concerns about subjects’ overestimate benefits and provide invalid informed consent | 174, 176, 200, 204, 205, 209 |
|  | confusions between research and therapy intensify extant problems of informed consent | 26, 31, 36, 40, 78, 174, 196, 201, 204, 205, 209 |
|  | should be clear that personal benefit does not overlap with the scientific purpose of the study | 9, 13, 89, 95, 117, 122, 209 |
|  | benefits to participants should be distinguished from benefits to society | 19, 174 |
|  | it is important to give very detailed information to patients participating in gene therapy trials to prevent unrealistic hopes | 170, 196 |
|  | risks should be communicated even if they are unlike to happen | 8, 12, 18, 46, 50, 51, 159, 160, 214 |
|  | gene therapy could be irreversible so the right to revoke one's consent is less meaningful than for continuing medical treatment | 50 |
|  | receiving insufficient information about the treatment is a main concern | 144 |
|  | participants prefer to wait for strong evidence before considering enrolling in a clinical trial | 8, 73, 86 |
|  | informed consent should inform participants, no protect the institutions | 151 |
|  | | |
| **Confidentiality** | difficulties in protecting the privacy and confidentiality | 4, 12, 36, 47, 64, 97, 162, 171, 187, 197, 198, 217 |
|  | information obtained during gene therapy trials may adversely affect individuals receiving treatment or their families | 50, 171, 187, 197, 198, 217 |
|  | | |
| **Review and monitoring** | somatic gene therapy arises similar ethical issues than other medical technologies/treatments | 4, 6, 12, 18, 19, 22, 28, 32, 37, 38, 41, 44, 50, 63, 65, 66, 69, 70, 71, 72, 73, 76, 77, 78, 85, 93, 94, 96, 100, 102, 105, 111, 114, 122, 124, 126, 128, 143, 158, 162, 168, 171, 173, 175, 178, 179, 181, 185, 191, 216 |
|  | no need for special evaluation of gene therapy protocol because it is similar to other biotechnologies | 100, 107, 173, 216 |
|  | there are specific bioethical implications for gene therapy and must be carefully considered | 5, 16, 20 |
|  | gene therapy has very specific and unique ethical complexities comparing to other medical practices | 2, 39, 46, 71, 90, 119, 190, 208 |
|  | need for public involve in the review and monitoring protocols | 127 |
|  | a worldwide accepted and controlled bioethics convention is need it for gene therapy | 126 |
|  | need for special evaluation and audit of protocols | 11, 35, 40, 45, 57, 62, 81, 100, 113, 118, 121, 124, 131, 150, 154, 159, 160, 188, 190, 192, 200, 202, 210, 213 |
|  | the ethical complexity of gene therapy should not be approach only with ethics committee | 2, 147, 151, 154, 158, 159, 160, 162 |
|  | the protocol should be strictly followed and any changes in the protocol should be documented | 110, 115, 62, 89, 115, 137, 145, 187, 188 |
|  | should be effective means of control and discipline after the protocol is approved | 162 |
|  | any adverse event must be reported | 46, 62, 89, 115, 145 |
|  | there is an obligation to avoid harm | 19, 40, 87 |
|  | security issues should not be confused with ethical issues | 32 |
|  | | |
| **Risk/benefit ratio** | should be treated as a conventional medical therapy in determining risk/benefit ratios | 85, 192 |
|  | beneficence hinges on the potential for net benefit in the whole population while doing minimal harm to the individual | 32, 81 |
|  | there could be subtle benefits of gene therapy | 88, 100, 125 |
|  | non-viral vectors could be safer but still not efficient | 17, 39, 62, 67, 70, 73, 131 |
|  | long term transgene expression is limited | 142 |
|  | need for a distinction between medical benefits and collateral benefits | 196 |
|  | difficulties in risk/benefit balance because the risks are uncertain and cannot be reduced to one utility | 176, 193 |
|  | difficulties in risk/benefit balance related to how potential social benefits should be balanced against individual risks | 196, 201 |
|  | difficult in balance benefits and risks compared to the burden and prognosis of the disease | 18, 34, 40, 41, 48, 63, 95, 100, 104, 114, 121, 125, 190 |
|  | probabilities and outcomes for adverse events relating to gene therapy are difficult to define | 7, 10, 18, 22, 40, 42, 51, 63, 67, 104, 114, 117, 165, 184, 190 |
|  | new materials have novel properties that may affect humans in unpredictable ways | 7, 16, 61, 63, 64, 70, 90 |
|  | could produce serious and/or irreversible side effects | 10, 17, 18, 23, 43, 50, 54, 60, 64, 69, 71, 85, 86, 88, 90, 100, 101, 114, 126, 167, 176, 183, 192 |
|  | could happen an unintentional modification of the germinal cells | 31, 54, 64, 67, 85, 88, 107, 114, 117, 125, 126, 164, 175, 177, 180, 202 |
|  | could produce immune responses generated against both the vector and the transgene | 54, 62, 118, 161, 164, 165, 168, 169, 175, 176, 177, 194, 196, 202, 213 |
|  | the gene vector could either activate an oncogene or inactivate a tumour-suppressor gene | 164, 169 |
|  | possible risks are transfer of an unwanted gene, administration of replication competent virus and bacterial contamination of the vector | 177, 196, 202 |
|  | concerns about the long-term safety and efficacy | 12, 16, 17, 31, 40, 41, 45, 59, 60, 61, 63, 64, 67, 69, 76, 77, 89, 90, 105, 123, 166, 175, 182 |
|  | technical issues in terms of the quality and stability of the transgene expression | 17, 31, 41, 59, 70, 85, 90, 110, 161, 168, 183, 184, 192, 196, 200, 202, 213 |
|  | viral vectors are still not quite safe | 17, 39, 61, 62, 64, 70, 71, 73, 90, 99, 100, 110, 118, 131, 142, 161, 165, 167, 183, 187, 196, 202, 213 |
|  | | |
| **Conflicts of interest** | difficulties in management of conflicts of interest | 33, 39, 40, 53, 77, 85, 93, 100, 102, 121, 124, 115, 145, 146, 188, 207, 213 |
|  | conflicts of interest could be financial and personal | 207 |
|  | important stakeholders have deep interests in gene therapy | 127, 155 |
|  | clinical investigators should not have personal financial relationship with companies that may benefit with results | 46 |
|  | due to the great investments, there is a big pressure for success on the scientists | 4, 53, 117, 121 |
|  | overlapping roles could lead to potential conflicts in the recruitment of subjects | 104 |
|  | | |
| **Legal regulations** | regulatory system is likely to be challenged by gene therapy | 6, 21, 22, 31, 45, 67, 66, 68, 69, 121, 136, 159, 160, 190, 201 |
|  | gene therapy research is highly regulated and is affected by overregulation and bureaucracy | 65, 68, 135, 179, 207 |
|  | gene therapy regulation cannot be a broad “blanket”, but each type needs to be assessed on its own merits and risk analysis | 149 |
|  | | |
| **Research priorities and limits** | gene therapy should be used in diseases evaluated in advance | 71, 85, 101, 125 |
|  | the boundaries for what should be the therapeutic objective have to be established | 175 |
|  | neither scientists or pharmaceutical companies should not control or decide alone about gene therapy limits | 4, 156 |
|  | if gene therapy would be determined by market forces, this would lead to the development of genetic technology for enhancement | 4, 191 |
|  | need to redefine rights and responsibilities of all actors involved | 14, 17, 109, 117, 150, 152, 155, 184, 210, 213 |
|  | human gene pools are a collective property, so a public debate is needed about gene therapy | 50 |
|  | the need for public participation in the ethical, social and policy discussion around gene therapy | 4, 50, 53, 58, 200 |
|  | could be difficult to design regulation considering political and cultural differences | 17, 62, 60, 63, 64, 68, 75, 76, 83, 85, 120, 127, 136, 152, 201 |
|  | it is no longer gene therapy per se being debated, but its application to particular diseases or particular patients | 179, 193, 216 |
|  | should be more efforts to prevent diseases rather than treat | 4 |
|  | gene therapy should not be a "first line" of defence therapy as long as an alternative is available | 18 |
|  | | |
| **Unproven use** | use of unproven gene therapy could apply to rare diseases | 3 |
|  | potentially high prices or limited availability of approved gene therapy may patients to seek unproven use | 215 |
|  | | |
| **Long term implications** | need to consider the long-term implications (specially the absence of vertical transmission) | 4, 154, 162, 164, 187 |
|  | need an adequate follow-up and to provide ongoing care for participants | 10, 22, 54 |
|  | several factors work against achieving follow-up of patients participating in gene therapy trials | 187 |

Table 2: Society-related arguments.

| **Category** | **Arguments** | **ID/s of article/s where the argument was/were extract** |
| --- | --- | --- |
| **Human identity** | human identity is under constant redefinition in biomedicine | 76, 91, 105 |
|  | humanity's identity is more than a pool of genes | 127, 216 |
|  | gene therapy could modify human identity, humanness, or personal perception | 11, 19, 27, 47, 69, 79, 101, 103, 109, 123, 131, 133, 191, 199, 212, 216 |
|  | effort is part of what makes us appreciate our lives, so we do not have to eliminate all the pain or suffering | 47 |
|  | we could lose our caring characteristics | 47 |
|  | could threaten human dignity | 208 |
|  | gene therapy involves causing particular human individuals to cease to exist | 4, 103 |
|  | the body could be perceived as an enemy or as a source of weakness perfectible by technology | 133 |
|  | gene therapy will reshape ideas on how best to live | 2 |
|  | gene therapy should not be used to change human traits | 162 |
|  | | |
| **Conceptual redefinitions** | there are no ethical differences between germline and somatic gene therapy | 25, 29 |
|  | we are not conceptually forced to allow all kinds of gene therapy once we allow one | 96 |
|  | biotechnology highlights moral problems but not creates them | 44 |
|  | research in somatic gene therapy cannot be considered eugenics | 172 |
|  | could create a need for a new disease/illness, prevention, and treatment concepts | 11, 14, 49, 81, 110, 113, 122,126, 133, 208 |
|  | enhancement or eugenic therapy could be captured as a therapy of human genetic disease | 167 |
|  | could be difficult to difference enhancement from treatment | 11, 14, 29, 44, 47, 64, 66, 72, 74, 80, 81, 85, 94, 96, 97, 101, 102, 109, 110, 113, 114, 120, 122, 126, 132, 179, 185 |
|  | | |
| **Disabilities and diverse functions** | is not necessary to overcome every human "limitation" | 4, 47, 79, 81, 83, 91, 103, 105 |
|  | disability could be an integrated aspect of a person’s identity | 133 |
|  | diverse functions or bodies that do not imply disabilities to prevent or treat (like deafness) | 47 |
|  | gene therapy could impact on the social attitudes on disability | 133 |
|  | the possibility of pursuing a better human could lead to more discrimination to disable people | 47 |
|  | gene therapy will not increase discrimination, it will make us aware of it | 6, 81 |
|  | instead of working on solutions based on social bias we need to think again about our social values | 47 |
|  | | |
| **Biodiversity concerns** | gene therapy will replace the animal tissue culture used in current treatments | 164 |
|  | there seems to be little concern in the impact of gene therapy on biodiversity | 4 |
|  | gene therapy manufacturing could be dangerous to the environment | 1, 136 |
|  | failure to treat ourselves as part of the environment of which we are part | 4 |
|  | | |
| **Population impact** | gene therapy research is a significant step on science evolution, and therefore, for humanity's well-being | 40, 65, 67, 69, 70, 72, 74, 76, 78, 83, 105, 106, 107, 118, 124, 126 |
|  | not affect human evolution | 165 |
|  | gene therapy of one person could have bad repercussions on others | 16, 27, 37, 44, 70, 77, 82, 85, 90, 93, 97, 114, 121, 126, 157, 200 |
|  | could modify human evolution | 37, 43, 76, 77, 81, 82, 91, 93, 94, 96, 101, 109, 122, 123, 126, 157, 167, 183, 184, 212, 217 |
|  | could increase the possibility of developing other new technologies with undesirable effects | 4, 35, 71, 72, 80, 93, 94, 96, 97, 101, 106, 122, 123, 128, 165, 183, 191, 199 |
|  | genetic diseases could become more prevalent in each generation after the somatic gene therapy | 37, 43, 202 |
|  | could eventually lead us to accept eugenic goals | 4, 49, 52, 74, 81, 85, 94, 96, 157, 172, 208, 217 |
|  | could lead us to modify the colour of the skin or change our personality based on social stereotypes | 167, 171 |
|  | new approaches have novel properties that may affect humans in unpredictable ways | 142 |
|  | longevity could provoke loneliness, and overpopulation, despite of improving quality of life | 1 |
|  | might cause harmful or unacceptable genetic alterations or lead to social abuses | 158 |
|  | could turn social problems into genetic problems | 4, 29, 85, 93 |
|  | gene therapy arises the issues of fairness, justice, or equity in access to therapy | 69, 67, 75, 81 |
|  | gene therapy could reduce personal privacy, lead to genetic discrimination, and cause population aging | 180 |
|  | if we accept somatic gene therapy, we are logically committed to accepting germ-line therapy | 44, 72, 122, 208 |
|  | the need to consider broad and long-range research consequences: the public health, environmental, and evolutionary concerns | 200, 201 |
|  | genetic technology is offered with the focus on individual patient choice | 70, 72, 79 |
|  | could motivates/deepen conflicts between values | 17, 35, 101, 107, 121, 152, 163 |
|  | "bad" genes are needed from the viewpoint of the species | 106 |
|  | | |
| **Social justice** | gene therapy could be cost-effective when compared with current therapies | 50, 53, 55, 69, 143, 162, 164, 189, 202, 215 |
|  | possibility of gene therapy reinforces the need for universal access to health care | 86, 197 |
|  | debates about genetics and justice should take seriously the fact of scarcity | 195, 197 |
|  | could be only available in countries/people with high income | 1, 14, 17, 21, 33, 34, 36, 76, 77, 79, 90, 96, 101, 102, 183, 189, 197 |
|  | could be discriminatory to people who do not have access to gene therapy | 11, 28, 36, 63, 81, 84, 101, 123, 185, 198, 212 |
|  | it may relegate funding from other areas of healthcare | 4, 21, 32, 34, 36, 38, 61, 64, 69, 83, 75, 77, 79, 85, 112, 119, 125, 197, 202 |
|  | economic inequities could impact human biology | 112 |
|  | | |
| **Public perception** | there is a high public support for the use of gene therapy to cure serious diseases but not to enhancement | 9, 19, 45, 50, 61, 63, 66, 67, 73, 74, 81, 85, 90, 97, 101, 106, 107, 113, 144, 167, 180, 212 |
|  | gene therapy is viewed by the majority as a desirable extension to the range of medical options available | 179 |
|  | in regard to therapeutic means, the Church is receptive and encouraging, so long as proper precautions are taken | 186, 198 |
|  | lay people are interested in knowing about gene therapy | 212 |
|  | guarantee sound research in general and patients’ safety in particular is crucial to public support and recruiting | 146 |
|  | ambivalence about genetic technology | 208 |
|  | gene therapy has a long way to go before gaining widespread acceptance among medical students | 180 |
|  | lay people think that is a risky procedure | 127 |
|  | there is no public trust in gene therapy | 4, 8, 127 |
|  | people are unaware of "gene therapy" term and its availability | 69, 86, 97, 126 |
|  | the possible consequences of manipulating genes or design humans arise fear | 9, 15, 60, 86, 93, 97, 98, 101, 105, 106, 126, 212 |
|  | the most frequent reasons for not accepting GT were fears of adverse effects, high cost and a belief that it went against nature | 180, 216 |
|  | concerns about the political uses of gene technology, genetic discrimination, and misuses of power | 180, 208 |
|  | genetic manipulation leads to a touchy issue about alteration of the soul, and therefore the Church wants to proceed slowly | 186 |
|  | could provoke negative emotional reactions because of the stories of deaths | 23, 62, 121, 131, 150, 163, 165, 210 |
|  | | |
| **Human health** | could be the only type of treatment for particular diseases | 11, 23, 31, 43, 50, 60, 62, 68, 70, 110, 111, 123, 128, 175, 179, 181, 182, 183, 185, 192 |
|  | has many potential applications, other than only in monogenetic diseases | 59, 62, 64, 69, 70, 73, 145, 161, 175, 181 |
|  | could prevent/treat serious diseases that make humanity suffer and improve quality of life | 60, 64, 68, 69, 73, 74, 80, 83, 84, 133, 140, 143, 169, 182, 185, 192, 199, 211 |
|  | progress in genetic research is clearly relevant to women's health for understanding and treating common diseases | 197 |
|  | “therapeutic abortion” could be rare if genetic diseases could be treated | 53, 129 |
|  | gene therapy could avoid anxiety associated with the life-threatening nature of the underlying disease | 53 |
|  | gene therapy also holds the promise of preventing diseases | 155 |
|  | gene therapy may provide a curative rather than a symptomatic approach to diseases | 143 |
|  | the treatment objective of gene therapy is not always curative, but rather aims at restoring function than eliminating the cause | 175 |
|  | there is a moral obligation to develop gene therapy if we consider it is the only treatment for particular diseases | 12, 19, 33, 36, 76, 125, 129, 194 |
|  | | |
| **Implementation** | gene therapy requires specific cooperation between healthcare workers and scientists | 64 |
|  | gene therapy will create a need for specific standard operational procedures | 64 |
|  | could set up problems in its implementation into the practice of medicine | 38, 59, 66, 67, 68, 131, 159, 165, 193, 194, 213 |
|  | genetic diagnoses are needed before the therapy, so it should be already available | 56, 81, 84, 123, 189 |
|  | analogous to present medical practices, therapeutic manipulation objectifies the disease in the person rather than the person | 217 |
|  | if alternative treatment exists, use of gene therapy will depend on its efficiency, costs, and level of discomfort to patients | 59 |
|  | | |
| **Communication to people** | the term "gene therapy" use in research does not reflect whether is a therapy or research | 50, 53, 54, 89, 93, 95, 104, 107, 113, 117, 124, 150, 161, 201, 204, 213 |
|  | terminology has been shown to influence risks and benefit perception | 205, 209 |
|  | public opinion should be adequately informed about gene therapy | 81 |
|  | scientists need to spend proper time in communicating science to media | 8, 137, 149, 212 |
|  | need for public trust on the basis on proper knowledge and transparency on research process | 14, 15, 17, 62, 68, 66, 81, 84, 90, 100, 108, 150, 152, 161, 163, 165, 184, 213 |
|  | there is a tendency to exaggerate potential benefits and minimize potential risks | 68, 66, 78, 124, 134, 190, 202, 216 |
|  | gene therapy research could evoke unrealistic expectations more than other areas of medical research | 66 |
|  | it is an emotionally volatile topic and if no patient is helped the negative reaction could provoke a society slowdown | 155, 169 |
|  | | |
| **Playing God** | we are not playing God with gene therapy as science is a human activity | 127 |
|  | there may be both proper and improper ways of playing God | 203 |
|  | humankind should not play God by doing gene therapy research | 76, 81, 91, 106, 122, 157, 167, 208 |
