# Supplementary material for: Bioethics of somatic gene therapy: what do we know so far?
Source: Curr Med Res Opin. Author manuscript; Available in PMC 2025 Jan 30. (PMC11780552; doi:10.1080/03007995.2023.2257600)

Appendix 6: Descriptive figures of the cohort of articles.

Figure 1. Numbers of articles per year.


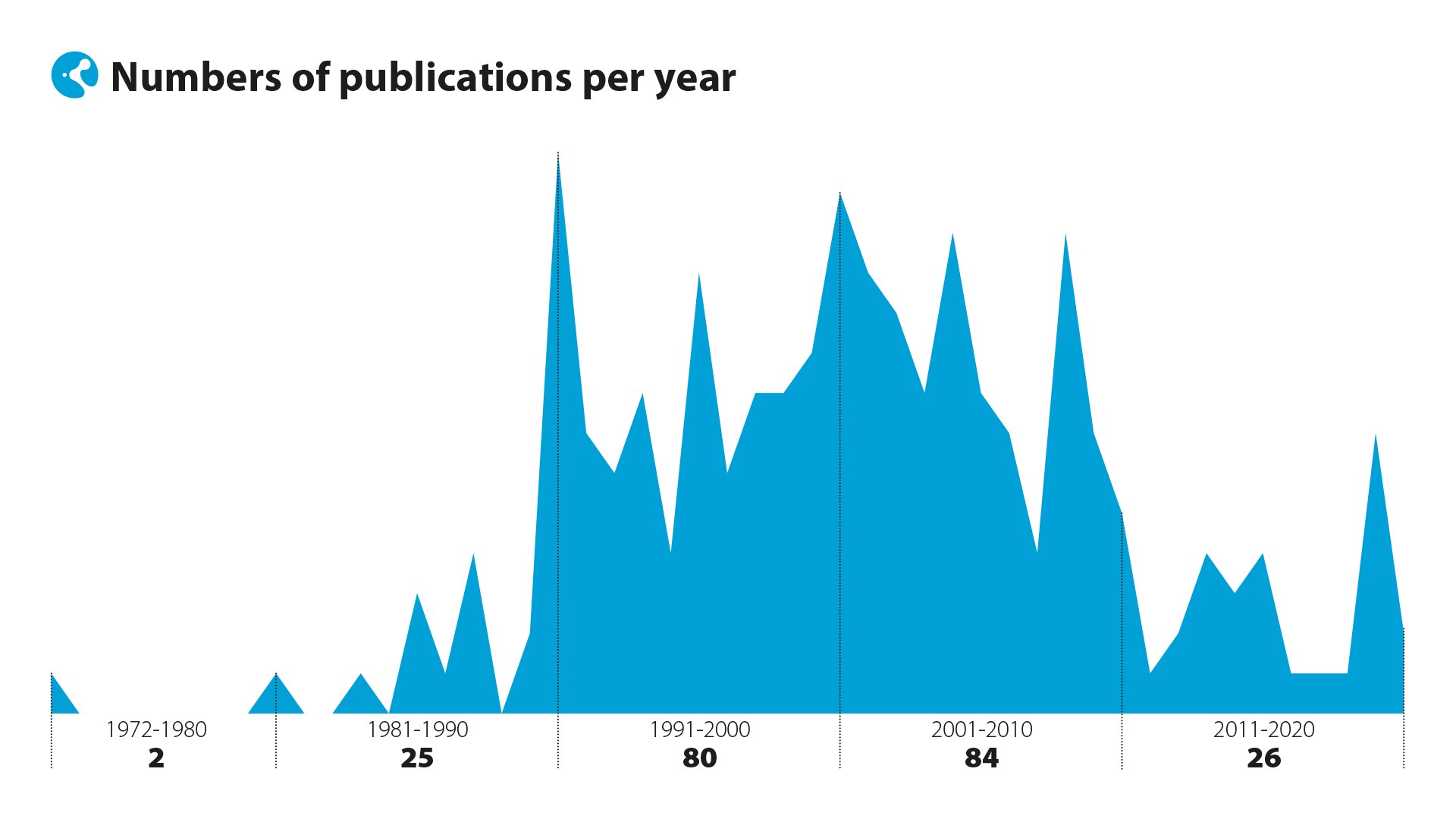


Figure 2. Types of articles.


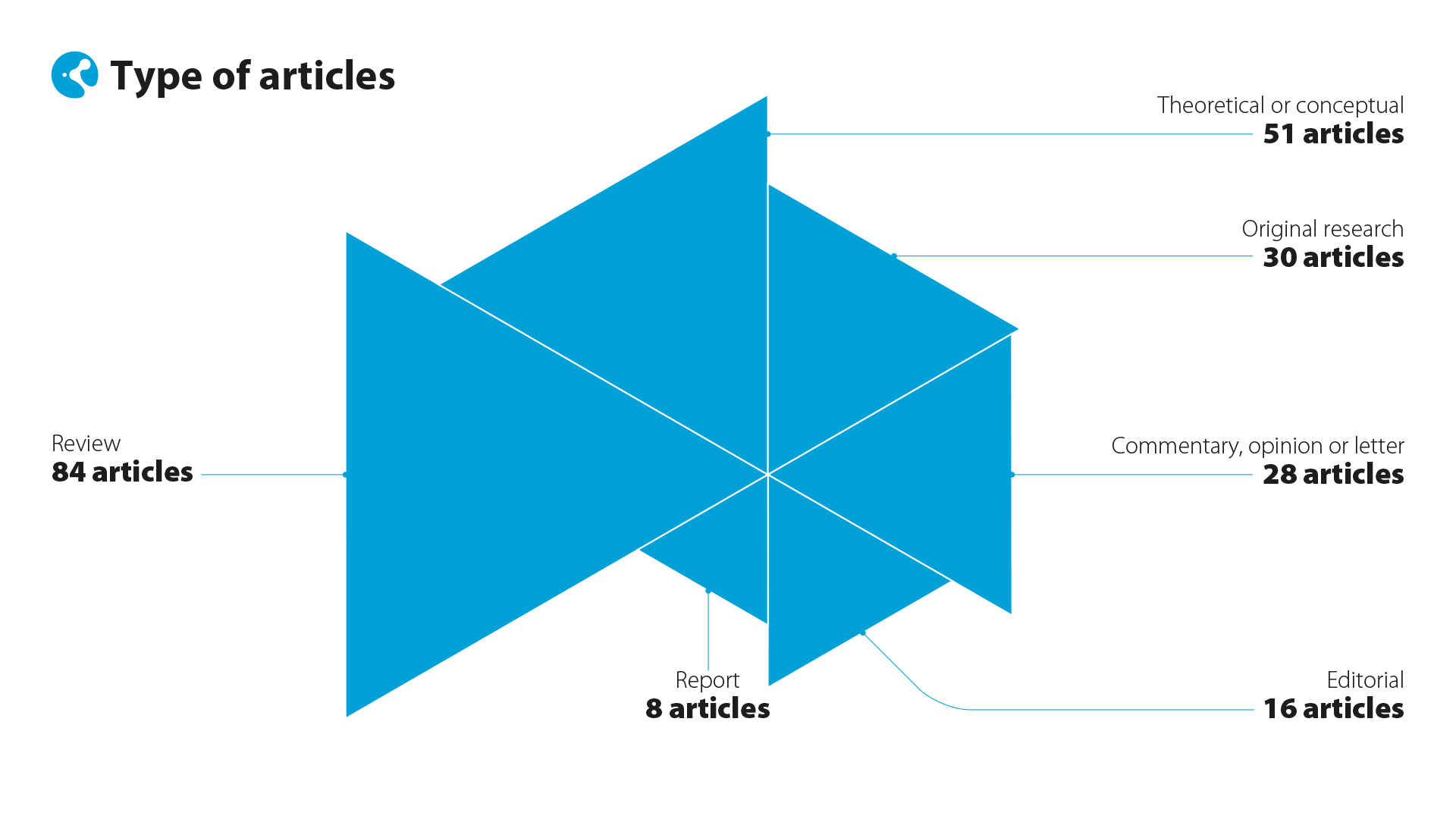


Figure 3. Country affiliation of all authors of included articles.


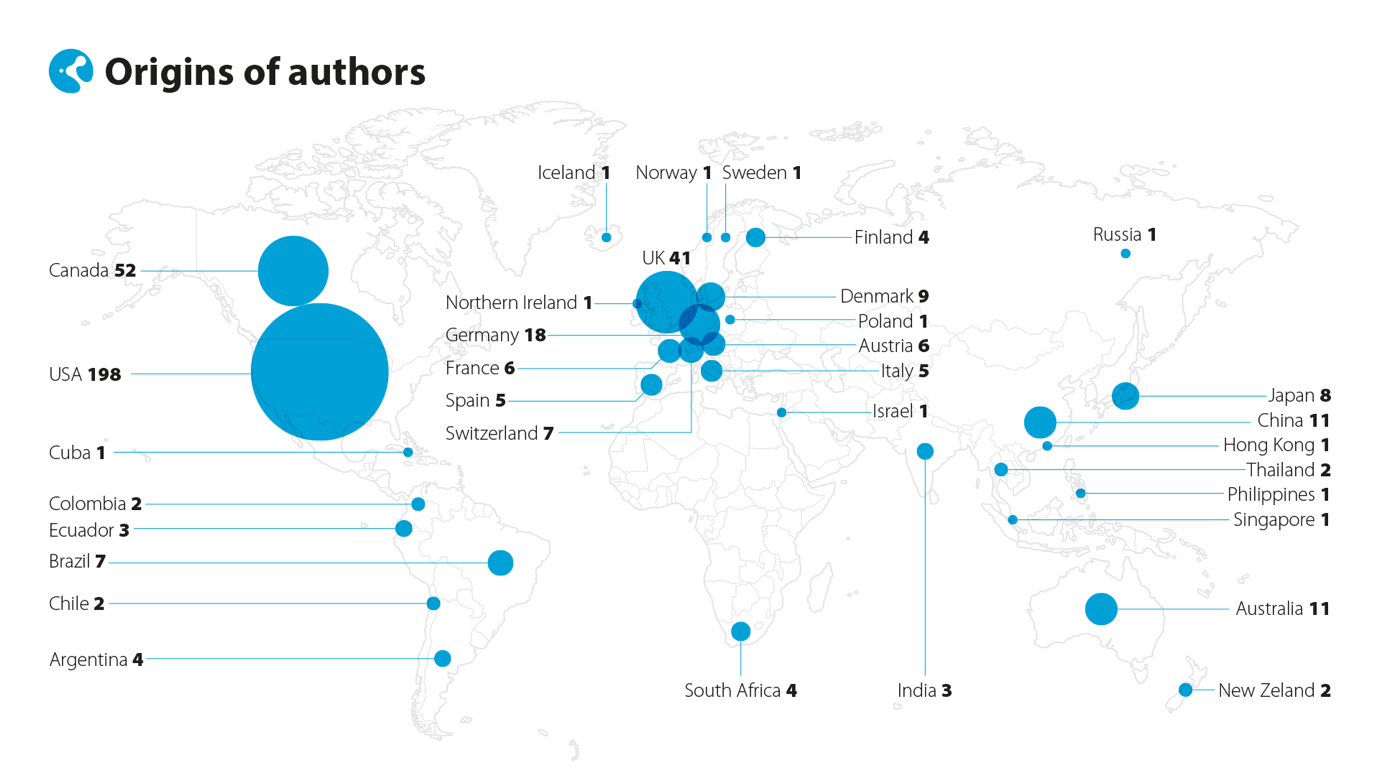


Figure 4. Journals in which articles have been published.


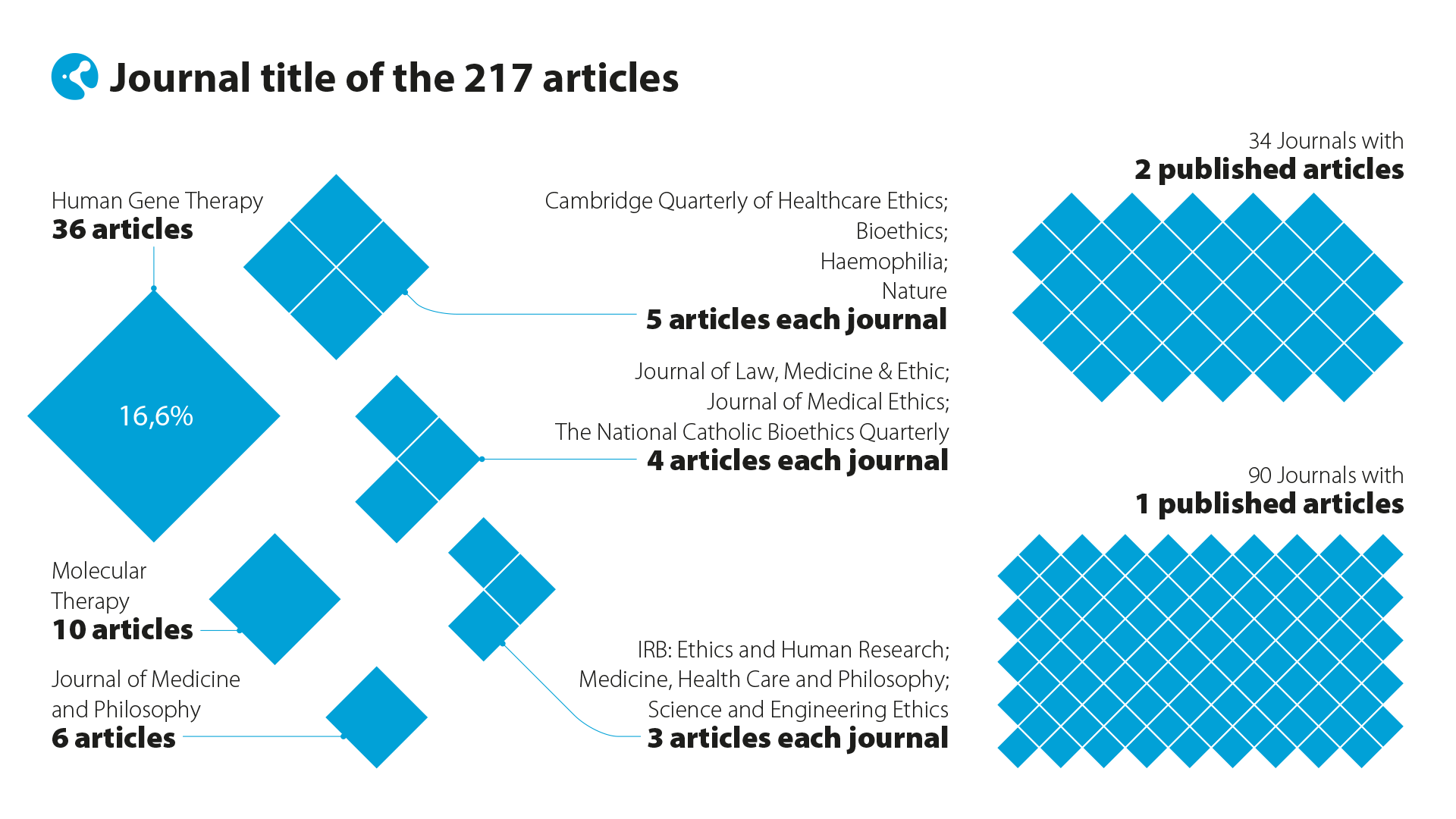


Figure 5. Academic fields (according to Journal Citation Report (JCR)) of the journals in which articles were published.


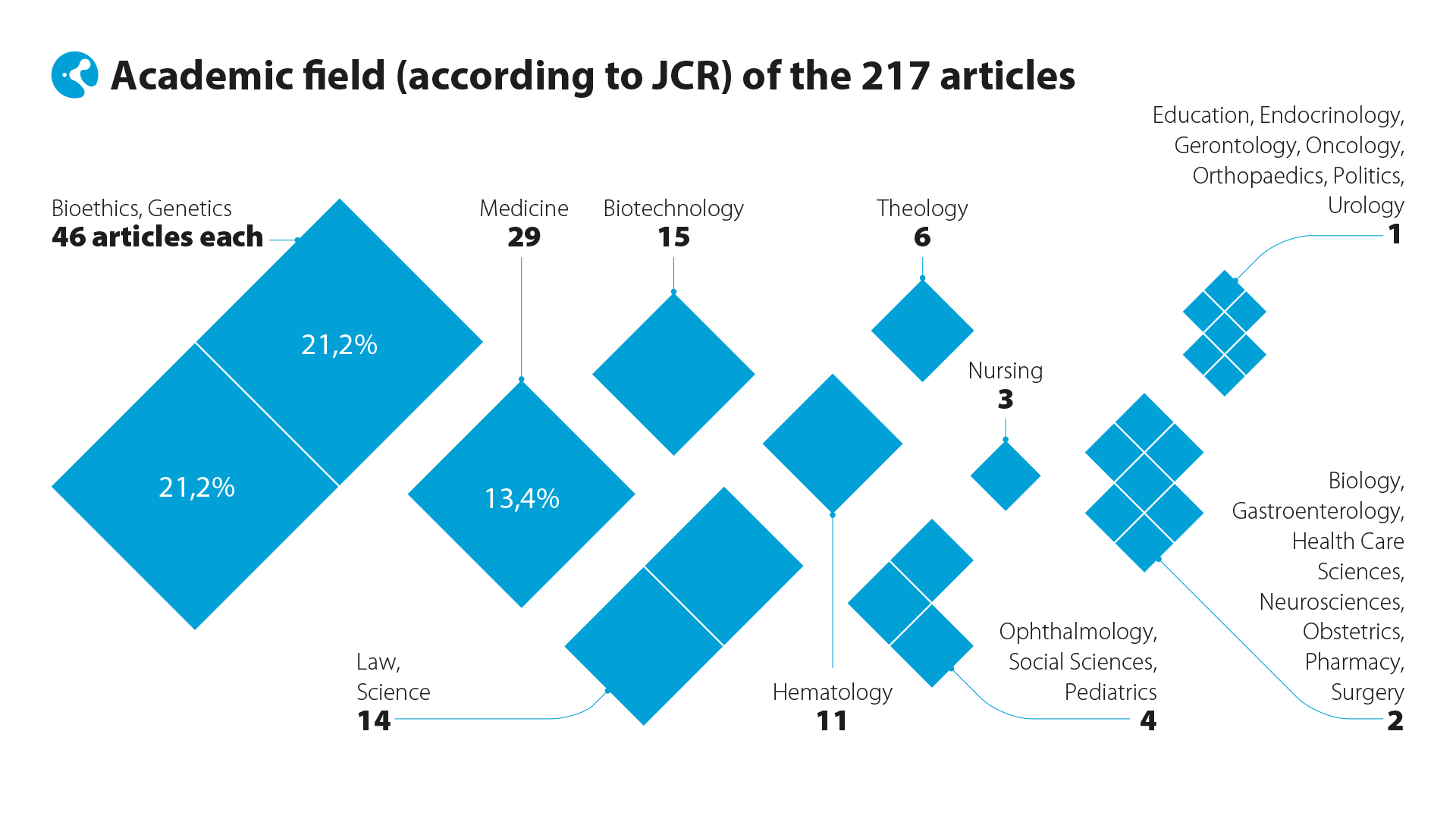

Supplement: Bioethics what Supp 6 [file NIHMS2040356-supplement-Bioethics_what_Supp_6.docx]
